# Uncovering the Differential Molecular Basis of Adaptive Diversity in Three *Echinochloa* Leaf Transcriptomes

**DOI:** 10.1371/journal.pone.0134419

**Published:** 2015-08-12

**Authors:** Gyoungju Nah, Ji-Hoon Im, Jin-Won Kim, Hae-Rim Park, Min-Jung Yook, Tae-Jin Yang, Albert J. Fischer, Do-Soon Kim

**Affiliations:** 1 Department of Plant Science, Research Institute of Agriculture and Life Sciences, College of Agriculture and Life Sciences, Seoul National University, Seoul 151–921, Korea; 2 Department of Plant Sciences, College of Agricultural and Environmental Sciences, University of California Davis, Davis, CA 95616, United States of America; Institute of Genetics and Developmental Biology, Chinese Academy of Sciences, CHINA

## Abstract

*Echinochloa* is a major weed that grows almost everywhere in farmed land. This high prevalence results from its high adaptability to various water conditions, including upland and paddy fields, and its ability to grow in a wide range of climates, ranging from tropical to temperate regions. Three *Echinochloa crus-galli* accessions (EC-SNU1, EC-SNU2, and EC-SNU3) collected in Korea have shown diversity in their responses to flooding, with EC-SNU1 exhibiting the greatest growth among three accessions. In the search for molecular components underlying adaptive diversity among the three *Echinochloa crus-galli* accessions, we performed *de novo* assembly of leaf transcriptomes and investigated the pattern of differentially expressed genes (DEGs). Although the overall composition of the three leaf transcriptomes was well-conserved, the gene expression patterns of particular gene ontology (GO) categories were notably different among the three accessions. Under non-submergence growing conditions, five protein categories (serine/threonine kinase, leucine-rich repeat kinase, signaling-related, glycoprotein, and glycosidase) were significantly (FDR, q < 0.05) enriched in up-regulated DEGs from EC-SNU1. These up-regulated DEGs include major components of signal transduction pathways, such as receptor-like kinase (RLK) and calcium-dependent protein kinase (CDPK) genes, as well as previously known abiotic stress-responsive genes. Our results therefore suggest that diversified gene expression regulation of upstream signaling components conferred the molecular basis of adaptive diversity in *Echinochloa crus-galli*.

## Introduction

The genus *Echinochloa* consists of approximately 50 species across tropical and warm temperate zones [1]. *Echinochloa* can grow in a broad range of habitats, and these plants are common weeds that have caused serious crop losses in rice and maize fields, leading to 32% to 99% reductions in crop yields [2–6]. In this genus, *Echinochloa oryzicola*, *E*. *crus-galli* var. *crus-galli*, *E*. *crus-galli* var. *praticola* have been major weeds in Korea [7,8]. Morphologically, the three species are barely indistinguishable, except for several traits, such as the presence of seed awn and the pattern of leaf growth. For example, a typical *E*. *oryzicola* has a relatively short seed awn compared to the long seed awn in *E*. *crus-galli* var. *crus-galli*. However, *E*. *crus-galli* var. *praticola* has no awn [8]. Cytologically, *E*. *oryzicola* is known to be tetraploid (2n = 4x = 36), whereas *E*. *crus-galli* is hexaploid (2n = 6x = 54) [9]. In eco-physiological traits, the three varieties are quite different. *Echinochloa oryzicola* grows only in flooded rice paddies, whereas *E*. *crus-galli* var. *praticola* mostly inhabits dry land and *E*. *crus-galli* var. *crus-galli* populates the areas from dry land to paddy rice fields [10]. Submergence tolerance of the three species has been systematically investigated using controlled environments [7]. In those studies, *E*. *oryzicola* was demonstrated to be the most submergence tolerant of the three species: The ranking for plant growth after 30 days of full (20 cm) submergence at the third leaf stage was *E*. *oryzicola* > *E*. *crus-galli* var. *crus-galli* > *E*. *crus-galli* var. *praticola*, supporting previous studies in the field [7].

One way to survive submergence stress is to change gene expression regulators, such as transcription factors. In submergence-tolerant species, certain transcription factors, such as the ethylene-responsive factor (ERF), have been demonstrated to play important roles in coping with submergence stress [11–14]. Under submergence (low O_2_ concentration), the ethylene content in plants increases and can either stimulate or repress the elongation of internodes through ERF induction [12–16], such as *SNORKEL1* (*SK1*) / *SNORKEL2* (*SK2*) [17] and *Submergence1* (*Sub1*) [18,19]. Another way to cope with abiotic environmental stimuli is through activating signal transduction pathways, which generate physiological responses for adaptation. Although there are multiple types of external stimuli, the signal transduction shares common features. The generic components of the signal transduction pathway for plant responses to abiotic stress include (1) a receptor kinase for signal perception, (2) second messengers for signal amplification, (3) an array of signal transduction components, and (4) transcription factors (TF, involved in stress-responsive gene expression) [20]. Transducers and TFs involved in signal transduction pathways that respond to cold [21–26], salinity [27–30], and drought [31–33] have been well-studied. On the contrary, the signal perception components have not been well-defined, as is the case for receptor-like kinases (RLKs), which have been known for their roles in disease resistance [34], and in developmental process [35]. Recently, the role of RLKs in abiotic stress tolerance has been reported in several plant species [36–42], but many RLKs in plants were still functionally unknown [43–45]. Global gene expression studies of RLKs in rice using microarray after stress treatment revealed that certain genes encoding RLKs are constitutively expressed in various tissues, suggesting that they have important roles in the regulation of drought, salt, and cold responses [43]. Therefore, RLKs may play important roles in abiotic stress signaling, in addition to their roles in development and biotic stress.

The presence of differential eco-physiology in the closely related species or within the same species would be a useful system to study adaptive variation. Presence of *Echinochloa* accessions in Korea within the same species that exhibit adaptive variation would serve such a purpose. In this work, we adopted the three accessions of *E*. *crus-galli* which exhibit adaptive variation to explore the molecular basis of such difference. Because genomic resources for *Echinochloa* species have been limited so far, largely due to its large genome size and polyploidy (2n = 4x = 36 or 2n = 6x = 54) [8], we used *de novo* transcriptome assembly as it has been an efficient approach for the large sized genomes of many crop species, saving cost and labor [46]. This approach was applied to two species of *Echinochloa* collected in China [47]. In this study, we investigated the composition and expression pattern of the three transcriptomes, in association with their ecological adaptive diversity. To identify major contributors, such as ERFs or RLKs, which are responsible for abiotic stress adaption, we tested for differential gene expression patterns and significant enrichment of protein categories in their leaf transcriptomes. Our results will serve as basis for differential adaptive diversity in *Echinochloa* and related grass species.

## Materials and Methods

### Ethics statement

No specific permits were required for the described field study. No specific permissions were required for this location and activities. The location is not privately owned or protected in any way. The field studies did not involve endangered or protected species.

### Plant materials and growth

Plants from three *E*. *crus-galli* accessions, EC-SNU1, EC-SNU2, and EC-SNU3, were collected in Seoul National University Experimental Farm Station in Suwon, Korea (E126°59'27.46'', N37°16'12.05'') in 2005. All *E*. *crus-galli* accessions were self-pollinated, and the progeny was processed for purification. One hundred seeds harvested from a single plant of each of the three *E*. *crus-galli* accessions that were selected based on parental characters for each accession, such as shape of seeds, existence of an awn and length of the outer glume (S1 Fig) [8]. The seeds were sown in petri dishes with wet filter paper and placed in a growth chamber (Hanbaek Scientific Ltd., Korea) under 25/20°C (day/night) and a 16 h photoperiod until the first leaf emergence. After the first leaf stage, twenty healthy seedlings of each *E*. *crus-galli* accession were selected and transplanted into to 11 cm diameter soil pots. Each *E*. *crus-galli* accession was isolated spatially to prevent contamination and allow it to grow to maturity. One plant per accession was selected as a typical morphological representative and its seeds were harvested. The seeds were stored at 4°C for 4 months to break their dormancy. Single-seed-descent lines were obtained by germinating seeds from each plant/species in Petri dishes and transplanting seedlings at the first leaf stage into pots filled with paddy soil; plants were grown in the glass house at the experimental farm station at Seoul National University, Suwon, Korea.

### Submergence treatment and growth measurement

Three accessions of *E*. *crus-galli* seeds were water-soaked in petri dishes in a growth chamber at a temperature of 30/25°C and a 16/8 h photoperiod (day/night) until the first leaf emerged. Germinated seedlings were transplanted in pots filled with well-puddled paddy soil in the glass house at the Experimental Farm Station of Seoul National University at Suwon, Korea. Plants at the third leaf stage were placed in acrylic boxes under three flooding depths or 0 cm (water-logging, control) or 20 cm (complete submergence) for up to 30 days. For gross morphology analysis, the measurements of plant height and photographic analyses were performed at 7 days after flooding. The height was examined from the top of the soil to the top of the main plant stem. The dry weight was investigated at 30 days after flooding. The plants were removed from the soil, dried in an oven set at 80°C for 48 hours and weighed on a scale. For plant height and dry weight, statistical analyses were performed using the IBM SPSS 21.0 software for Windows. Data were analyzed with analysis of variance (ANOVA) and the means were considered to be significantly different if the Tukey-HSD post-hoc test showed p < 0.05.

### Cytological analyses

For DNA content measurement, we performed flow cytometric analysis of the three *E*. *crus-galli* accessions using one sorghum accession (*Sorghum bicolor* cv. Pioneer 8695) as an internal standard, and one *E*. *oryzicola* accession as a tetraploid control. Leaf tissues (~0.5 cm^2^) were chopped with a double-edge razor in a petri dish containing 0.5 mL nuclear extraction buffer (CyStain UV precise P/Solution A, PARTEC, Germany). After samples were chopped, they were filtered through a 30 μm mesh filter. After filtration, 2.0 mL of a staining solution containing 4′,6′-diamidino-2-phenylindole (DAPI) fluorochrome (CyStain UV precise P/Solution B, PARTEC, Germany) was added. Flow cytometry was performed using Ploidy Analyzer (CyFlow PA, PARTEC, Germany). Fluorescence peaks were estimated using the WinMDI software package (version 2.9, http://en.bio-soft.net/other/WinMDI.html). Three replications per leaf were analyzed.

For chromosome counting, young root tips (~0.5 cm) were sampled and placed into 1.5 ml microtubes filled with 0.002 M 8-hydrozyquinolin. After 2 hours, the solutions were removed 3:1 absolute alcohol:glacial acetic acid was added at room temperature for one day. The fixed samples were washed with distilled water for 2 minutes, hydrolyzed with 5N HCl for 45 minutes and the root tips were rinsed with distilled with water for 2 min. The samples were stained using Schiff's stain (0.01 M pararosaniline hydrochloride, 0.01 M Potassium disulfite, 0.15N HCl) for 2 hours in darkness. After staining, samples were washed with distilled water for 10 minutes. The root tips were soaked in enzyme solution (cellulysin and macerase in 10^-3^M EDTA) for 45 minutes, placed on a slide glass and tapped with a stainless steel stick. The samples were observed under 1,000X magnification using a Photo Microscope (Axiophot, ZEISS, Germany).

### Phylogenetic analysis

*matK* and *rbcL* sequences from six species (*E*. *crus-galli* EC-SNU1, EC-SNU2, and EC-SNU3 accessions, *E*. *oryzicola*, *Oryza sativa*, and *Sorghum bicolor*) were used for phylogenetic tree construction and estimation of divergence time. The *matK* and *rbcL* sequences of three *E*. *crus-galli* accessions were retrieved from the previous chloroplast genome sequencing of three *E*. *crus-galli* accessions (Nah et al., unpublished), whereas the sequences of *E*. *oryzicola* (GenBank ID: KJ000048) [48], *O*. *sativa* (GenBank ID: NC_001320.1) and *S*. *bicolor* (GenBank ID: NC_008602.1) were obtained from GenBank (http://www.ncbi.nlm.nih.gov/gene). Multiple sequence alignment of *matK* cDNA sequences was performed using Clustal W (http://www.clustal.org/clustal2/). After sequence alignment, a phylogenetic tree was constructed using the BEAST package (http://beast2.org/) with a Hasegawa-Kishino-Yano (HKY) model of evolution used for Bayesian MCMC analysis. The tree was displayed through FigTree (version 1.4.2, http://beast.bio.ed.ac.uk/figtree). *matK* and *rbcL* sequences from the three *E*. *crus-galli* accessions were deposited into NCBI GenBank (EC-SNU1_*rbcL*: KR058322; EC-SNU2_*rbcL*: KR058323; EC-SNU3_*rbcL*: KR058324; EC-SNU1_*matK*: KR058325; EC-SNU2_*matK*: KR058326; EC-SNU3_*matK*: KR058327).

### RNA isolation, cDNA library construction, and sequencing

Total RNA was extracted from the fourth leaves of three *E*. *crus-galli* accessions before the first tillering, using Trizol reagents (Invitrogen, USA) according to the manufacturer's instructions. Quantity and quality of total RNA were analyzed using a Nanodrop 2000 spectrophotometer (Nanodrop, USA) and gel electrophoresis (Takara, Japan), respectively. cDNA library construction was performed using the TruSeq kit (Illumina, USA), at the National Instrumentation Center for Environment Management (NICEM), at Seoul National University, Korea. Sequencing was performed by 101bpX2 (paired-end sequencing) for Illumina HiSeq2500 (Illumina, CA, USA) at NICEM. All reads were deposited in the National Center for Biotechnology Information (NCBI) center under the SRA accession of SRP041999.

### *De novo* assembly, annotation, and gene ontology categorization

For *de novo* contig assembly, a total of 78,500,009 paired end reads were obtained in three libraries; the EC-SNU1 library has 22,713,977 paired reads, the EC-SNU2 library has 29,405,121 paired reads, and the EC-SNU3 library has 26,380,911 paired reads. We filtered the raw reads in two steps: the low quality reads were removed by the trimming script from the FASTX toolkit (version 0.0.12; http://hannonlab.cshl.edu/fastx_toolkit/). Reads with q-values less than 30 were trimmed, and then PCR duplicates were removed by the trimming script from the NGS QC toolkit (version 2.3; http://59.163.192.90:8080/ngsqctoolkit/). We used Trinity (r2013-02-25; http://trinityrnaseq.sourceforge.net/) for *de novo* assembly of the *E*. *crus-galli* leaf transcriptome reads (min_kmer_cov = 6, JM = 20G, CPU = 2, min_contig_size = 300). After assembly, we performed CD-hit (http://www.bioinformatics.org/cd-hit/) with an identity of 95% to remove redundancy. Next, we filtered out known transposable elements from the assembled contigs using mipsREdat_9.3p_ALL.fasta (http://www.mmnt.net/db/0/0/ftp.mips.embnet.org/plants/REdat) using BLASTN with an e-value less than 1E^-10^, an alignment identity greater than 90%, and an alignment length greater than 100bp. Annotation was performed using BLASTX with an e-value less than 1E^-5^ against *Oryza sativa* peptide sequence data with annotation information from Rice Genome Annotation Project (ftp://ftp.plantbiology.msu.edu/pub/data/Eukaryotic_Projects/o_sativa/annotation_dbs/ pseudomolecules/version_7.0/all.dir/all.pep). For gene ontology (GO) categorization, we used DAVID (http://david.abcc.ncifcrf.gov/), followed by calculation of relative ratios. The relative ratio was estimated using the number of contigs assigned to an individual GO category divided by the total number of contigs used for GO assignment for each accession.

### Identification of differentially expressed genes (DEG), hierarchical clustering, and GO-enrichment assay

Using assembled contigs from EC-SNU1 as a reference, we mapped raw reads from SRP041999 to quantify the expression level for DEG identification. For this purpose, run_DE_analysis.pl from edgeR (with q < 0.05) implemented in Trinityrnaseq_r2013-02-25 package was used. This script identified DEGs in pair-wise comparisons using mapped reads. Next, we calculated the FPKM (Fragments per Kilo base-pair per Million mapped fragments) value for individual genes in EC-SNU1, EC-SNU2, and EC-SNU3 using run_RSEM_align_n_estimate.pl from the Trinityrnaseq_r2013-02-25 package. FPKMs, when found with a value greater than 2 in at least one of the accessions, were used for gene expression clustering. For gene expression clustering, we used Pearson's correlation with average distance at GENE-E (http://www.broadinstitute.org/cancer/software/GENE-E/download.html) using the log_2_ value of FPKM. For the GO enrichment assay, we used DAVID (http://david.abcc.ncifcrf.gov/). We first selected contigs with significant matches to the Arabidopsis protein database (TAIR10) and 18,596 contigs matched with TAIR10 (e-value <1E^-10^) were used as a background gene set for DAVID. Contigs from DEG were converted to TAIR10 protein IDs based on their homologies and run as a sample gene set for DAVID. To select significant GO categories, we applied the q-value approach using a False Discovery Rate (FDR) threshold of less than 0.05. All other runs, such as BLASTN and BLASTX were performed using the LINUX operating system (CentOS, http://www.centos.org/download/).

### Quantitative real time PCR verification

For qRT-PCR analysis, leaf samples were collected from the full growth of 4^th^ leaf stage. Total RNA was extracted from 100 mg of leaf tissue using the RNeasy Plant Mini Kit (Qiagen, CA, USA). Single-stranded cDNA was synthesized from 500 ng of total RNA using TOP-script Reverse Transcriptase (Enzynomics, Korea). *eEF-1α* was used as a housekeeping gene to normalize the three *E*. *crus-galli* accessions. Primers for 19 genes (2 Serine/threonine receptor kinases, 4 Leucine-rich repeat receptor kinases, 5 Cysteine-rich protein kinases, and 8 Calcineurin B-like kinases) were used (S1 Table). qRT-PCR reactions were carried out using TOP-realTM qPCR 2X PreMIX for SYBR Green (Enzynomics, Korea) with a final volume of 20 μl in a Rotor-Gene Q 2plex real-time PCR machine (Qiagen, CA, US). The qRT-PCR cycle program was as follows: (1) 95°C for 15 min for initial denaturation, (2) 95°C for 10 sec for denaturation, (3) 55°C for 15 sec for annealing, (4) 72°C for 20 sec for elongation, and then the cycles from (2)-(4) were repeated 45 times. Relative quantification of each single gene expression was evaluated using the delta-delta Ct method [49].

## Results

### Morphological, cytological, and physiological overview of three *E*. *crus-galli* accessions

We investigated seed morphology of three *E*. *crus-galli* accessions, particularly, seed size and awn (S1 Fig). EC-SNU1 resembled more to the *E*. *oryzicola* than to *E*. *crus-galli*, but still exhibited relatively smaller size and longer awn than those of *E*. *oryzicola*. The seed shape of EC-SNU1 resembled the typical C-type *E*. *oryzicola* [50], but cytological analysis below revealed hexaploidy nature (S2 and S3 Figs), a major characteristic of *E*. *crus-galli*. EC-SNU2 also exhibited smaller seed size and the longest awn among all accessions, which are typical traits of *E*. *crus-galli* var. *crus-galli*. Finally, EC-SNU3 displayed in smallest seed size without awn, the typical traits of *E*. *crus-galli* var. *praticola*.

To obtain basic genetic information on these three *Echinochloa* accessions, we investigated their ploidy levels. Flow cytometry measurements showed that all three accessions carry DNA content (ANOVA, p < 0.05) similar to hexaploids (S2 Fig) Chromosome counting finally confirmed that they are hexaploids (2n = 6x = 54) (S3 Fig). This property is a major characteristic of *E*. *crus-galli*, which distinguishes it clearly from the tetraploid *E*. *oryzicola* (S2 and S3 Figs).

To validate the eco-physiological implications of the fact that EC-SNU1 and EC-SNU2 were collected from paddy rice field, whereas EC-SNU3 was collected from dry land, we investigated the physiological responses for the three accessions under controlled submergence conditions, and evaluated their growth by measuring their leaf growth and biomass yield. Compared to control (0 cm) and the full submergence (20 cm) treatment gave significant differences in growth among the three accessions, particularly EC-SNU1 exhibited significantly (p < 0.05) more growth compared to the other two accessions (S4 Fig).

### *De novo* assembly of three *E*. *crus-galli* leaf transcriptomes

**Fig 1:**
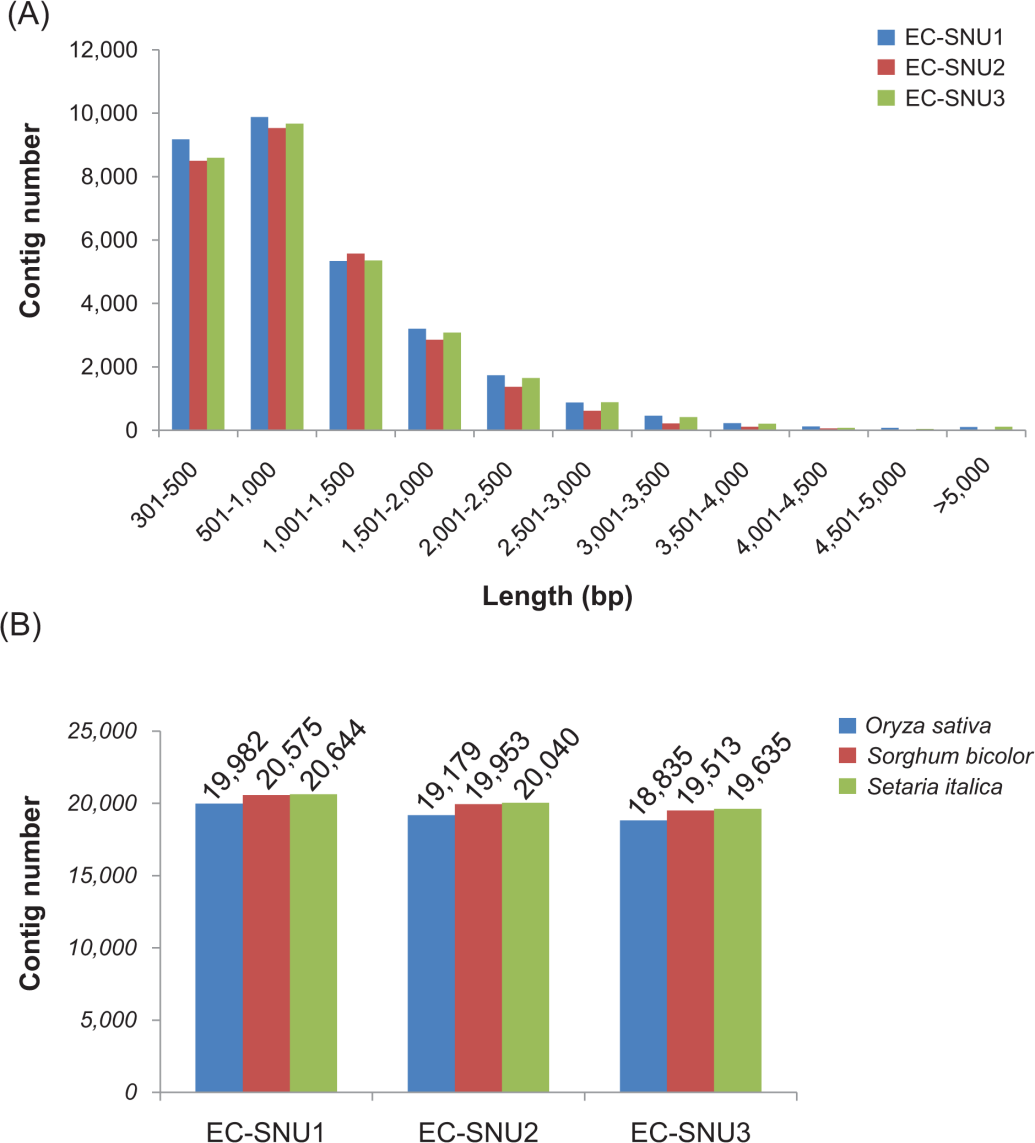
(A) Contig size distribution of *de novo* assembly of transcriptomes from three *Echinochloa* accessions. (B) Assembly validation of known grass protein databases for rice (*Oryza sativa*), sorghum (*Sorghum bicolor*), and foxtail (*Setaria italica*).

To investigate the genomic constituent of the three *E*. *crus-galli* accessions, we performed *de novo* assembly of raw reads from three *E*. *crus-galli* leaf transcriptomes generated by Illumina HiSeq. Initially, we obtained 18,368,735 reads from EC-SNU1 (81% recovery ratio), 23,428,201 reads from EC-SNU2 (80% recovery ratio), and 21,122,046 reads from EC-SNU3 (80% recovery ratio). Using Trinity with 25-mers, as recommended by the published procedure [51], we obtained 18,368,735 reads from EC-SNU1 (81% recovery ratio), 23,428,201 reads from EC-SNU2 (80% recovery ratio), and 21,122,046 reads from EC-SNU3 (80% recovery ratio). As a result, we retrieved 31,160 contigs with N50 of 1,435 bp from EC-SNU1, 28,814 contigs with N50 of 1,278 bp from EC-SNU2, and 30,048 contigs with N50 of 1,411 bp from EC-SNU3 (Table 1). Because we used cDNA fragments sequenced by paired-end (101bpX2) reading with a minimum of 100 bp between the two ends, 300 bp was chosen as the minimum contig size. As a result, the average length of a contig was 1,050 bp, 976bp and 1,049bp in EC-SNU1, EC-SNU2, and EC-SNU3, respectively (Table 1). The range of contig sizes showed that the majority of contigs were 300–1,500bp, covering 78.2% (24,361 contigs) of the EC-SNU1 library, 81.8% (23,565 contigs) of the EC-SNU2 library, and 78.5% (23,585 contigs) of the EC-SNU3 library (Fig 1A).

**Table 1:** Statistics of *de novo* assembly of *Echinochloa* leaf transcriptomes.

	EC-SNU1	EC-SNU2	EC-SNU3
Total number of contigs	31,023	28,882	30,048
Total bases (bp)	32,586,794	31,527,271	31,527,271
Minimum contig length (bp)	301	301	301
Maximum contig length (bp)	12,060	7,412	11,788
Average contig length (bp)	1,050	977	1,049
N_50_ length (bp)	1,435	1,278	1,411

### Annotation and gene ontology categorization of three *E*. *crus-galli* leaf transcriptomes

For assembly validation and annotation, BLASTX against known protein databases of *Oryza sativa*, *Sorghum bicolor*, and *Setaria italica* was performed. Using a threshold e-value of less than E^-05^ and an aligned amino acid length of 50aa, 63–67% of the translated *E*. *crus-galli* contigs were matched against the *Oryza sativa* protein database, 65–69% against the *Sorghum bicolor* protein database, and 65–70% against *Setaria italica* protein database (Fig 1B). In all three accessions, the C_4_ grass *Setaria italica* showed the highest match among three grass species protein databases. Next, the annotated contigs were subjected to gene ontology (GO) categorization for three categories (biological, cellular, and molecular process), and the three accessions showed similar relative gene ratios of genes in each individual category, and exhibited the same categories with similar ratios of contigs in the leaf transcriptomes (S5 Fig, S2, S3, and S4 Tables). The top five categories were also well-conserved among the three accessions: For biological process, the top five categories included biological process (12.7–13.6% of an individual transcriptome), cellular process (12.0–12.2%), metabolic process (11.0–11.3%), biosynthetic process (7.5–7.9%), and response to stress (4.7–4.9%). For cellular process, cellular component (13.8–14.6%), plastid (13.0–13.7%), membrane (11.7–12.4%), plasma membrane (8.6–8.9%), and nucleus (8.4–8.6%) were included in the top five. For molecular process, molecular function (17.2–18.5%), catalytic activity (10.8–11.1%), protein binding (10.2–10.8%), hydrolase activity (8.5–8.7%), and DNA binding (8.0–8.8%) were included in the top five. Overall, GO categories and their relative ratios were well conserved among the three leaf transcriptomes. To investigate the homolog ratios shared by three leaf transcriptomes, we performed pair-wise BLASTN with an e-value of less than 1E^-10^. The majority of the transcriptome consisted of homologs, ranging from 80.4% to 92.2% (S6 Fig).

### Differential gene expression of three *E*. *crus-galli* leaf transcriptomes

**Fig 2:**
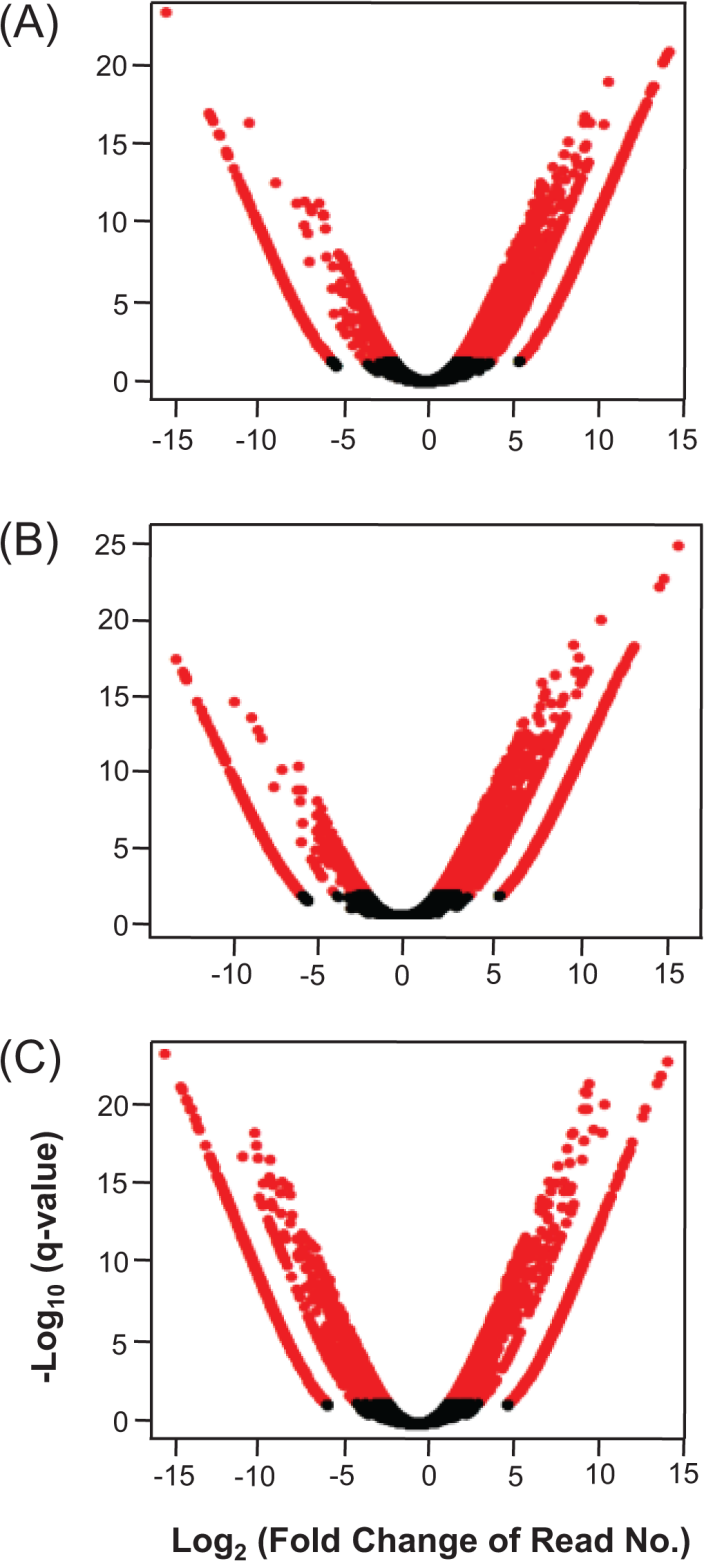
Distribution of log_2_ fold change of DEGs in pair-wise comparison; (A) EC-SNU1 vs. EC-SNU2, (B) EC-ENU1 vs. EC-ENU3, and (C) EC-SNU2 vs. EC-SNU3.

Although EC-SNU1 and EC-SNU2 showed no significant physiological and growth difference in submergence response (S4 Fig), it is possible that they are different at molecular level in relation to other abiotic stress. To perform systematic investigation of overall abiotic stress-related gene expression in three accessions, we conducted pair-wise comparisons of the DEGs and detected the presence of significant (q < 0.05) differential gene expression. The pattern of DEG distribution exhibited a bias toward up-regulation in the EC-SNU1 contigs in all pair-wise comparisons (Fig 2A and 2B). However, the comparisons for EC-SNU2 and EC-SNU3 exhibited a relatively similar number of contigs in the DEG distributions (Fig 2C). We counted the total number of contigs belonging to these DEGs. In each pair-wise comparison, 1,061 contigs were up-regulated in EC-SNU1 compared to 399 up-regulated in EC-SNU2, 936 were up-regulated in EC-SNU1 compared to 269 up-regulated in EC-SNU3, and 857 were up-regulated in EC-SNU2 compared to 756 up-regulated in EC-SNU3 (S7 Fig).

**Fig 3:**
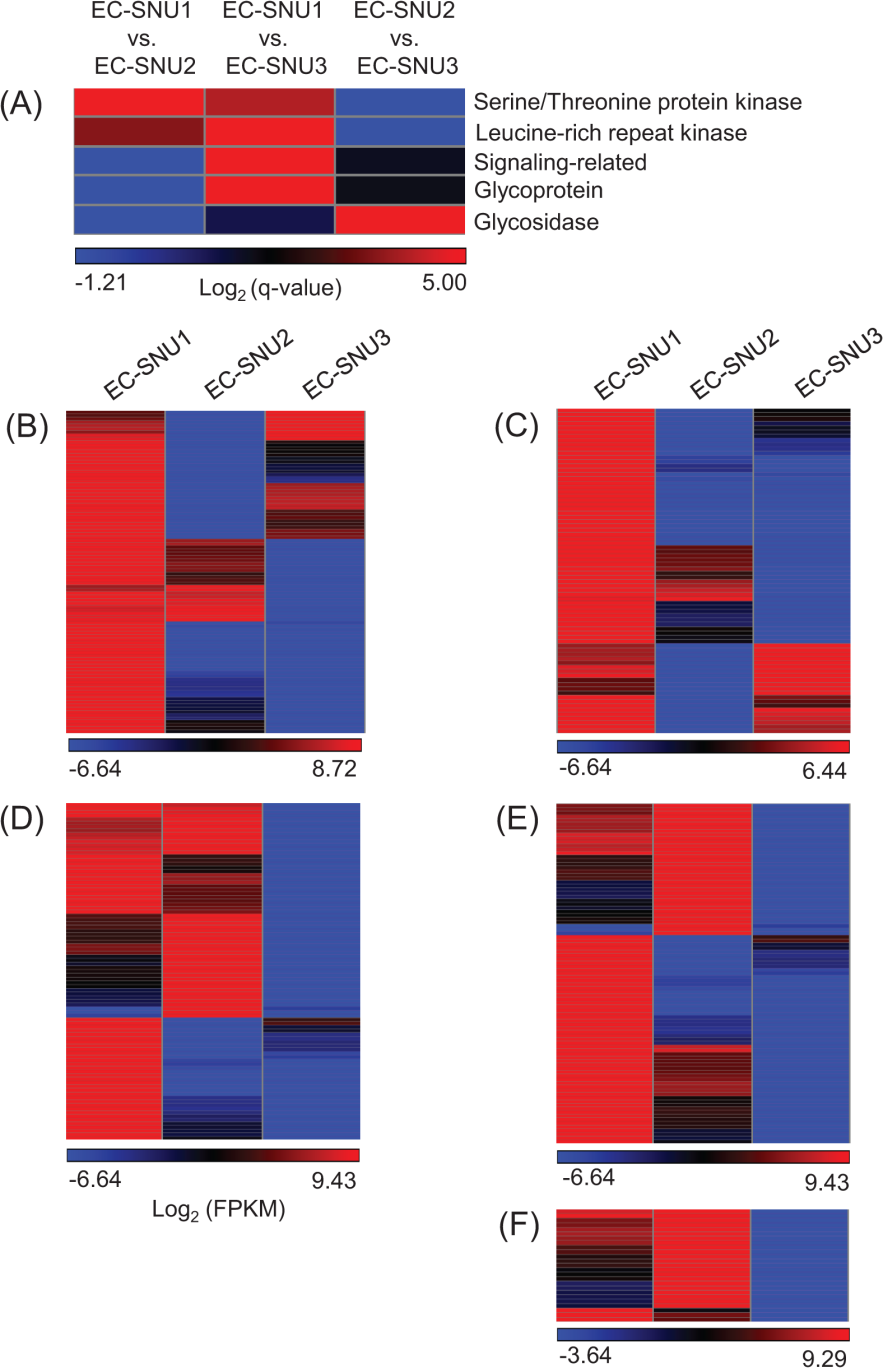
(A) Significant GO category enrichment in pair-wise comparisons. (B) Hierarchical clustering of DEG belonging to serine/threonine protein kinases. (C) Hierarchical clustering of DEG belonging to leucine-rich repeat kinases. (D) Hierarchical clustering of DEG belonging to signaling-related proteins. (E) Hierarchical clustering of DEG belonging to glycoprotein. (F) Hierarchical clustering of DEG belonging to glycosidase.

To examine whether a large number of up-regulated DEGs are enriched with particular protein categories, GO enrichment analysis was performed. Five groups of representative enriched protein categories from SWISS-PROT and the Protein Information Resource Database were retrieved: (1) serine/threonine protein kinase (STK) (GO:0004674), (2) leucine-rich repeat receptor-like kinase (LRR-RLK) (GO:0035872), (3) signaling-related protein (GO:0023052), (4) glycoprotein (GO:0047965), and (5) glycosidase (GO:0016798) (Fig 3A). A hierarchical clustering diagram of STK and LRR-RLK groups of annotated transcripts were significantly up-regulated in EC-SNU1 relative to both EC-SNU2 and EC-SNU3 (Fig 3B and 3C, S5 and S6 Tables). Although there was up-regulation of DEGs of EC-SNU2 and EC-SNU3 against those of EC-SNU1 in STK and LRR-RLK groups (Fig 3B and 3C), majority of up-regulation of DEGs was enriched in EC-SNU1. Of the RLKs, particularly Ca^2+^-related signaling kinases were identified with dominant number in EC-SNU1 (S8A Fig, S7 Table), which are known participants in signal transduction. A hierarchical clustering diagram of signaling-related and glycoprotein groups of annotated transcripts were significantly up-regulated in both EC-SNU1 and EC-SNU2, relative to EC-SNU3 (Fig 3D and 3E, S8 and S9 Tables). Finally, a hierarchical clustering diagram of glycosidase group of annotated transcripts was significantly up-regulated in EC-SNU2, relative to EC-SNU1 and EC-SNU3 (Fig 3F, S10 Table). In addition to five GO categories, we observed the homologs of previously known abiotic stress-related genes in Arabidopsis were identified as DEGs and outnumbered in EC-SNU1, compared to EC-SNU2 and EC-SNU3 (S8B Fig, S11 Table).

**Fig 4:**
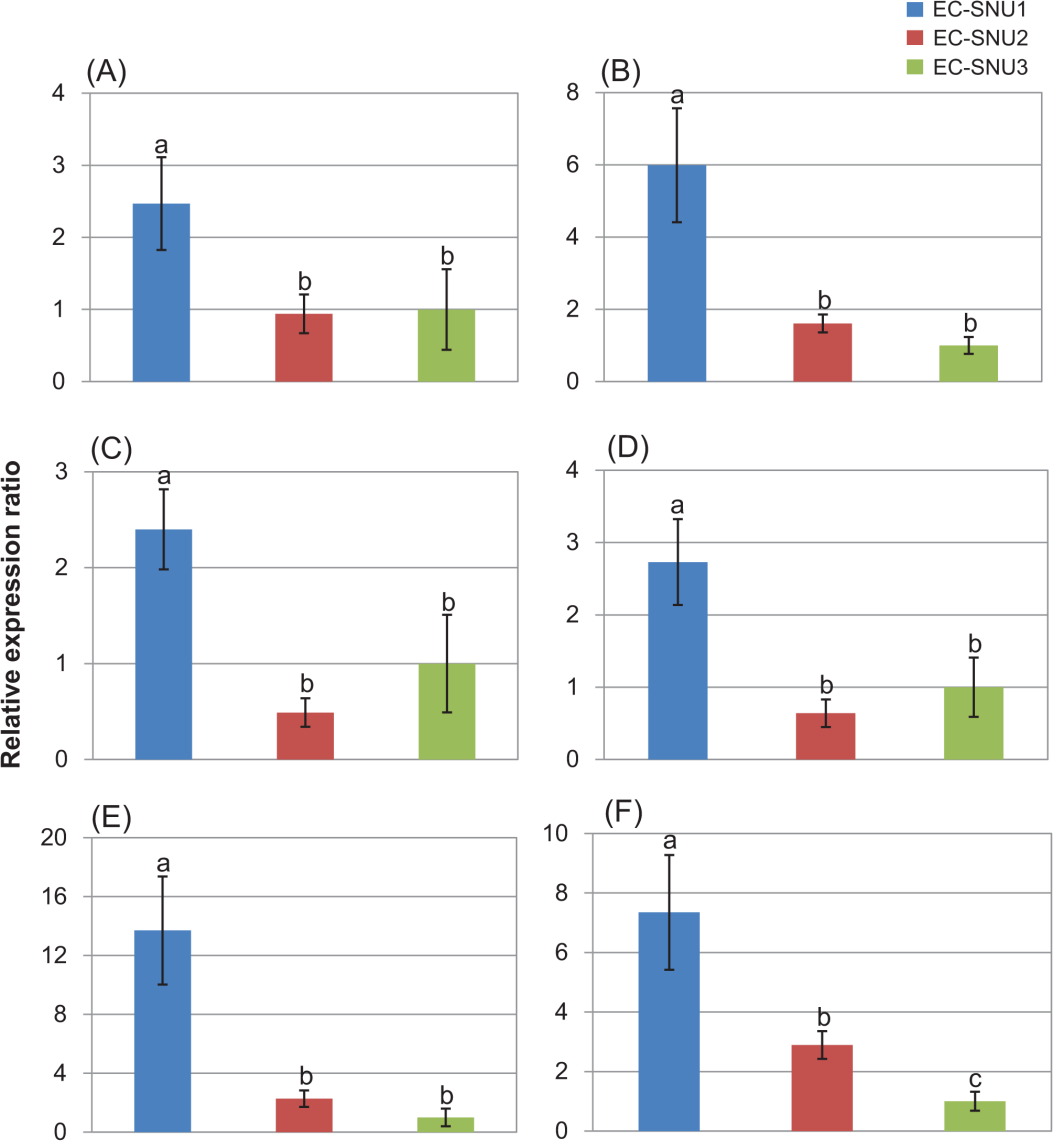
qRT-PCR validation of significantly up-regulated DEGs in EC-SNU1;(A) SOS3-interacting protein 1, (B) Calcium-dependent kinase 6, (C) Calmodulin 1, (D) LRR-RLK, (E) Cysteine-rich RLK 29, and (F) Cysteine-rich RLK 40.

We were particularly interested in RLK and 19 randomly selected DEGs were validated for relative expression using qRT-PCR (S1 Table), and of them, six genes were displayed (Fig 4). In most cases of *RLK* genes, the relative expression ratio was highest in EC-SNU1 (Fig 4).

We also investigated known submergence tolerance genes, *SNORKEL1/2* (*SK1/2*) [17] and *Submergnece1A* (*Sub1A*) [18], in our transcriptomes based on sequence homology, but we could not detect any homologs of *Sub1A/B/C* or *SK1/2*. Instead, we observed several *ERF* homologs that were up-regulated in EC-SNU1 when EC-SNU1 was compared to EC-SNU3, and in EC-SNU2 when EC-SNU2 was compared to EC-SNU3. In both cases, the homolog of AT1G28370 (ERF domain 11), from the B-1 subfamily of the ERF/AP2 transcription factor family, was observed. An association between AT1G28370 and abiotic stress has been reported in previous studies [52–54].

### Phylogenetic relationship among the three *E*. *crus-galli* accessions

The five enriched GO protein categories were detected in a step-wise manner in pair-wise comparisons, such that enrichment of up-regulated glycosidase category was found in EC-SNU2 against EC-SNU3 and EC-SNU1, enrichment of up-regulated of the signaling-related and glycoprotein categories were found in EC-SNU1 and EC-SNU2 against EC-SNU3, and finally, enrichment of up-regulated serine/threonine kinase and LRR kinase, categories were found in EC-SNU1 against EC-SNU2 and EC-SNU3 (Fig 3A). This observation raised the question as to whether such a step-wise acquisition supports our current understanding of the evolutionary divergence order. To answer this question, we established the phylogenetic relationship among the three accessions using two chloroplast genes, *matK* and *rbcL*. We used the *matK* and *rbcL* genes because they have been known as the most rapidly evolving coding regions in the chloroplast genome, and their sequence diversity provides a high level of discrimination to establish evolutionary relationships among angiosperm species [55]. Thus, these two genes have been selected to establish phylogenetic relationship by the Consortium for the Barcode of Life (http://www.barcoding.si.edu/plant_working_group.html). Evolutionary relationship from the phylogenetic trees using both *matK* and *rbcL*, suggested that EC-SNU1 diverged from the most recent common ancestor of EC-SNU2 and EC-SNU3, followed by split into EC-SNU2 and EC-SNU3 (S9 Fig).

## Discussion

Based on transcriptome sequence homology against protein databases using BLASTX, the C4 plant *Echinochloa* was best matched against another C4 species, foxtail millet (*Setaria italica*). Foxtail millet is a close relative of C4 bioenergy crops, such as switchgrass (*Panicum virgatum*), napiergrass (*Pennisetum purpureum*), and pearl millet (*Pennisetum glaucum*) [56], and its whole genome was sequenced [57], serving as a useful C4 reference system for other C4 grass species [58]. With foxtail millet, 60~70% of transcripts of *Echinochloa* were significantly matched, indicating the conservation of common genetic components among these C4 grasses. At the same time, the detection of 30~40% of difference in the transcriptome indicates that they might be mis-assembled contigs or *Echinochloa*-specific transcripts.

Although EC-SNU1 and EC-SNU2 showed no significant difference in flooding response based on flooding treatment experiment, they are still useful system together with EC-SNU3 in the comparative aspect, because they exhibited the variation at gene expression level in relation to abiotic stresses. Interestingly, we found that DEGs of EC-SNU1 significantly up-regulated, compared to those of EC-SNU2 and EC-SNU3, at gene expression level of certain abiotic-stress related GO categories, particularly RLK genes. To cope with unfavorable environmental changes, plants need to develop dynamic endogenous mechanisms, such as signal transduction. RLKs have been known to play important roles in sensing abiotic stress and signaling to the downstream network [59–62]. RLK forms a large gene family, consisting of over 600 genes in *Arabidopsis* [45,63] and over 1,000 genes in rice [45], indicating active and diverse roles in signaling from the environment stimuli. RLK-encoding genes are known to be increased by tandem duplication, and the presence of a large number of paralogs provides a source for genetic diversity [44,64,65]. Previous studies have demonstrated that RLKs act as key regulators in various abiotic stress responses in many plant species, such as *Arabidopsis thaliana* [66–69], *Oryza sativa* [41,70,71], *Medicago truncatula* [36], and *Glycine soja* [72]. These abiotic stress-related RLKs include leucine-rich repeat receptor-like protein kinases (LRR-RLK) [38,73], cysteine-rich RLK (CRK) [66], and calcium/calmodulin-dependent kinase (CaMK) [66], all of which were found as DEGs in our analysis. One important feature of RLKs is that they are upstream components in signal transduction. The significant up-regulation of RLK-encoding genes in EC-SNU1 reflected that this accession might have evolved in a different way within *E*. *crus-galli* via modification of regulatory regions. Although we tested only submergence, EC-SNU1 might have acquired additional abiotic stress tolerance traits, a hypothesis that needs further testing. This result suggests that evolutionary processes selected one or more upstream genes in the signaling pathway, probably because this conferred more efficient and dramatic impacts on the downstream pathways.

Moreover, our results suggested that expression pattern change might be an efficient strategy for plants to cope with abiotic stress, rather than generating new genes. Although it is possible that transcription of RLK-encoding genes can also be induced by external stimuli, constitutive up-regulation, as seen in the tolerant accession, can be a strategy for rapidly coping with adverse environmental conditions. For example, the gene expression profiling of 17 calcium-dependent protein kinase (CDPK) genes in grapevine exhibited constitutive expression that is prevalent in pollen [74], indicating that constitutive expression is one of the adaptive signaling strategies. Therefore, adaptive diversity can be achieved by inducing differential expression of signaling genes via modification of their regulatory regions. Moreover, previous studies indicated that modification of gene expression patterns can enhance abiotic tolerance. For example, the study on RLK showed that water-stress related tolerance was enhanced in *RPK1*-overexpressing plants, whereas loss-of-function mutants showed down-regulation of stress-related genes [68]. Additionally, rice *OsSIK1*-overexpressing plants exhibited enhanced salt and drought tolerance, whereas loss of function of *OsSIK1* resulted in sensitivity to these stresses [37].

**Fig 5:**
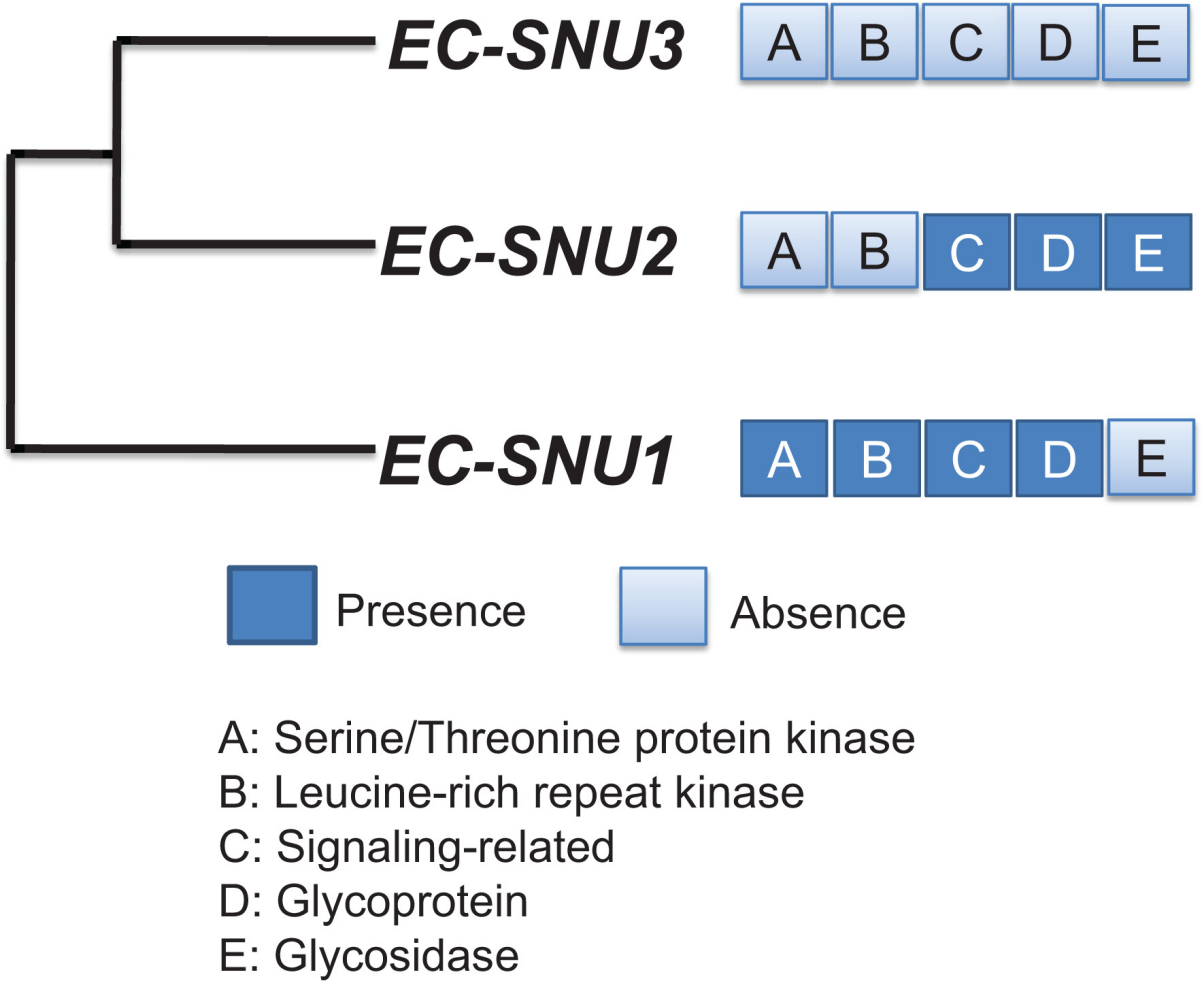
The step-wise change of gene expression pattern in the phylogenetic relationship of three *E*. *crus-galli* accessions.

Another observation is that the step-wise loss and acquisition of gene expression groups corresponded to the phylogenetic relationship of the three accessions (S8 Fig). After divergence from EC-SNU1, the common ancestor of EC-SNU2 and EC-SNU3 appears to have lost gene expression up-regulation in two GO categories (STK and LRR-RLK), and then EC-SNU3 alone has lost up-regulation in two additional GO categories (signaling-related and glycoprotein). On the contrary, up-regulation of glycosidase appears to be acquired in EC-SNU2 after divergence from EC-SNU3. Based on this observation, we generated a model for the stepwise loss and acquisition of expression diversification in five GO categories during evolution (Fig 5). The availability of regulatory sequences for all three accessions will be helpful to validate this result by determining the diversification of the regulatory regions.

Unlike the RLKs, we were unable to detect orthologs of both *Sub1A/B/C* and *SK1/2* in three *E*. *crus-galli* leaf transcriptomes. The absence of *Sub1* or *SK* in *E*. *crus-galli* may be due to the limitations of the transcriptome size, or because *E*. *crus-galli* does not carry these genes. Instead, we identified one *ERF* homolog (AT1G28370) in the DEGs. This *E*. *crus-galli ERF* homolog might be the candidate for a submergence-responsive *ERF*. In addition, *ERF* is known to respond to drought, salt, and freezing stresses, indicating that *ERF*s might regulate multiple abiotic stresses [75]. Therefore, it may be useful to regulate multiple abiotic stress pathways using a single gene, such as genes encoding ERF or RLK, to generate stress-tolerant plants. We also identified new groups of proteins, glycoprotein and glycosidase, which might be related to abiotic stress tolerance. Although a very few studies have been done, recent reports provided involvedness of these groups of proteins in abiotic stress tolerance, by modifying cell wall metabolism [76–80].

Based on our analyses, we conclude that significantly up-regulated DEGs in EC-SNU1, including RLK-encoding genes, can be useful source for future studies of adaptive diversity, as well as for identification of novel abiotic stress-related genes in other grass species.

## Conclusions

The three *E*. *crus-galli* leaf transcriptomes were generated and served as a useful system to identify and explore the molecular basis for environmental adaptability among accessions. Comparison of the three accessions revealed that family of RLK-encoding genes, which has been known to play important roles in signal transduction in both biotic and abiotic stresses, were major contributors to adaptive diversity. Moreover, previously known abiotic stress-related genes were also observed in these *Echinochloa* leaf transcriptomes. The fact that RLK-encoding genes and previously known abiotic stress-related genes were significantly up-regulated primarily in EC-SNU1 compared to EC-SNU2 or EC-SNU3 suggests the presence of variation in environmental adaptability, even within the *E*. *crus-galli* species. The phylogenetic relationship among the three accessions also implies that the regulatory changes in many RLK-encoding genes have been maintained in EC-SNU1, whereas some of them were lost in EC-SNU2 and EC-SNU3 during evolution. Our analysis therefore implies that gene expression modification of signaling components plays an important role in adaptive diversity in *Echinochloa* accessions.

## Supporting Information

S1 Fig Seed morphology of *Echinochloa* accessions. (A) *E*. *oryzicola*, (B) EC-SNU1, (C) EC-SNU2, and (D) EC-SNU3.
(PDF)

S2 Fig Flow cytometric measurement of DNA content in *E*. *oryzicol*a and three *E*. *crus-galli* accessions; (A) *E*. *oryzicola* (tetraploid control), (B) EC-SNU1, (C) EC-SNU2, and (D) EC-SNU3. (PDF)

S3 Fig Chromosomes from *E*. *oryzicola* and three *E*. *crus-galli* accessions; (A) *E*. *oryzicola* (tetraploid control), (B) EC-SNU1, (C) EC-SNU2, and (D) EC-SNU3. (PDF)

S4 Fig Relative plant height (A) and relative dry weight (B) of *Echinochloa* accessions at 7 days after submergence treatment. (PDF)

S5 Fig GO categorization of assembled contigs from three *Echinochloa* leaf transcriptomes; (A) Biological process, (B) Cellular process, and (C) Molecular process. (PDF)

S6 Fig Pair-wise BLASTN of three *Echinochloa* transcriptomes. (PDF)

S7 Fig Contig number for significantly up-regulated DEGs based on pair-wise comparisons. (PDF)

S8 Fig (A) Hierarchical clustering of DEG belonging to CDPKs. (B) Hierarchical clustering of DEG belonging to previously known *Arabidopsis* abiotic sress responsive genes. (PDF)

S9 Fig Phylogenetic relationships among three *E*. *crus-galli* accessions in comparison with *E*. *oryzicola*, rice (*Oryza sativa*), and sorghum (*Sorghum bicolor*); (A) Phylogenetic relationship using *matK* genes and (B) Phylogenetic relationship using *rbcL* genes. (PDF)

S1 Table Primers used for qRT-PCR validation of selected DEGs. (DOCX)

S2 Table GO categorization for molecular process using three *E*. *crus-galli* transcriptomes. (DOCX)

S3 Table GO categorization for cellular process using three *E*. *crus-galli* transcriptomes. (DOCX)

S4 Table GO categorization for biological process using three *E*. *crus-galli* transcriptomes. (DOCX)

S5 Table Hierarchical clustering of DEGs belonging to serine/threonine protein kinases (STKs). (DOCX)

S6 Table Hierarchical clustering of DEGs belonging to leucine-rich repeat protein kinase (LRR-STKs). (DOCX)

S7 Table Hierarchical clustering of DEGs belonging to calcium-related signaling. (DOCX)

S8 Table Hierarchical clustering of DEGs belonging to signaling-related proteins. (DOCX)

S9 Table Hierarchical clustering of DEGs belonging to glycoproteins. (DOCX)

S10 Table Hierarchical clustering of DEGs belonging to glycosidases. (DOCX)

S11 Table Hierarchical clustering of DEGs belonging to previously known *Arabidopsis* abiotic stress-responsive genes. (DOCX)

## References

[1] Michael PW, editor. Flora of north America and north Mexico New York: Oxford University Press; 2003 pp. 390–403.

[2] Stauber LG, Smith RJ, Talbert RE. Density and spatial interference of barnyardgrass (*Echinochloa crus-galli*) with rice (*Oryza sativa*). Weed Sci. 1991;39: 163–168.

[3] Chauhan BS, Johnson DE. Implications of narrow crop row spacing and delayed *Echinochloa colona* and *Echinochloa crus-galli* emergence for weed growth and crop yield loss in aerobic rice. Field Crop Res. 2010;117:177–182.

[4] Moon BC CS, Kwon OD, Lee SG, Lee BW, Kim DS. Modelling rice competition with *Echinochloa crus-galli* and *Eleocharis kuroguwai* in transplanted rice cultivation. J Crop Sci Biotechnol. 2010;13: 121–126.

[5] Mennan H, Ngouajio M, Sahin M, Isik D, Altop EK. Competitiveness of rice (*Oryza sativa* L.) cultivars against *Echinochloa crus-galli* (L.) *Beauv*. in water-seeded production systems. Crop Prot. 2012;41: 1–9.

[6] Moon BC KJ, Cho SH, Park JE, Song JS, Kim DS. Modelling the effects of herbicide dose and weed density on rice-weed competition. Weed Res. 2014;54: 484–491.

[7] Kim DS. Characterization of emergence and early growth of *Echinochloa* spp. and rice under the condition of direct-seeding rice culture. M.Sc. Thesis, Seoul National University. 1993. pp. 29–46. Available: http://www.riss.kr/search/detail/DetailView.do?p_mat_type=be54d9b8bc7cdb09&control_no=83d84fcbfd298d43&outLink=N

[8] Lee EJ. Phylogenetic relationships of *Echinochloa* species based on phenotypic and SSRs markers. M.Sc. Thesis, Seoul National University. 2013. pp. 1–7. Available: http://dcollection.snu.ac.kr/jsp/common/DcLoOrgPer.jsp?sItemId=000000010442

[9] Gould FW, Ali MA, Fairbrothers DE. A revision of *Echinochloa* in the United States. Am Midl Nat. 1972;87: 36–59.

[10] Yamasue Y, Asai Y, Ueki K, Kusanagi T. Anaerobic seed germination for the habitat segregation in *Echinochloa* weeds. Jpn J Breed. 1989;39: 159–168.

[11] Jackson M. Ethylene-promoted elongation: an adaptation to submergence stress. Ann Bot. 2008;101: 229–248. 17956854 10.1093/aob/mcm237 PMC2711016

[12] Shang JZ, Song PH, Ma B, Qi XW, Zeng QW, Xiang Z, et al Identification of the mulberry genes involved in ethylene biosynthesis and signaling pathways and the expression of *MaERF-B2-1* and *MaERF-B2-2* in the response to flooding stress. Funct Integr Genomic. 2014;14: 767–777. 10.1007/s10142-014-0403-2 PMC4233114 25231943

[13] Jisha V, Dampanaboina L, Vadassery J, Mithofer A, Kappara S, Ramanan R. Overexpression of an AP2/ERF type transcription factor *OsEREBP1* confers biotic and abiotic stress tolerance in rice. PLoS One. 2015;10: e0127831 10.1371/journal.pone.0127831 26035591 PMC4452794

[14] Bui LT, Giuntoli B, Kosmacz M, Parlanti S, Licausi F. Constitutively expressed *ERF-VII* transcription factors redundantly activate the core anaerobic response in *Arabidopsis thaliana* . Plant Sci. 2015;236: 37–43. 10.1016/j.plantsci.2015.03.008 26025519

[15] Papdi C, Perez-Salamo I, Joseph MP, Giuntoli B, Bogre L, Koncz C, et al The low oxygen, oxidative and osmotic stress responses synergistically act through the ethylene response factor VII genes *RAP2*.*12*, *RAP2*.2 and *RAP2*.*3* . Plant J. 2015;82: 772–784. 10.1111/tpj.12848 25847219

[16] Qi W, Sun F, Wang Q, Chen M, Huang Y, Feng YQ, et al Rice ethylene-response AP2/ERF factor OsEATB restricts internode elongation by down-regulating a gibberellin biosynthetic gene. Plant Physiol. 2011;157: 216–228. 10.1104/pp.111.179945 21753115 PMC3165871

[17] Hattori Y, Nagai K, Furukawa S, Song XJ, Kawano R, Sakakibara H, et al The ethylene response factors SNORKEL1 and SNORKEL2 allow rice to adapt to deep water. Nature. 2009;460: 1026–1030. 10.1038/nature08258 19693083

[18] Xu K, Xu X, Fukao T, Canlas P, Maghirang-Rodriguez R, Heuer S, et al *Sub1A* is an ethylene-response-factor-like gene that confers submergence tolerance to rice. Nature. 2006;442: 705–708. 16900200 10.1038/nature04920

[19] Fukao T, Bailey-Serres J. Submergence tolerance conferred by *Sub1A* is mediated by SLR1 and SLRL1 restriction of gibberellin responses in rice. Proc Natl Acad Sci U S A. 2008;105: 16814–16819. 10.1073/pnas.0807821105 18936491 PMC2575502

[20] Huang GT, Ma SL, Bai LP, Zhang L, Ma H, Jia P, et al Signal transduction during cold, salt, and drought stresses in plants. Mol Biol Rep. 2012;39: 969–987. 10.1007/s11033-011-0823-1 21573796

[21] Carvallo MA, Pino MT, Jeknic Z, Zou C, Doherty CJ, Shiu SH, et al A comparison of the low temperature transcriptomes and CBF regulons of three plant species that differ in freezing tolerance: *Solanum commersonii*, *Solanum tuberosum*, and *Arabidopsis thaliana* . J Exp Bot. 2011;62: 3807–3819. 10.1093/jxb/err066 21511909 PMC3134341

[22] Kim Y, Park S, Gilmour SJ, Thomashow MF. Roles of CAMTA transcription factors and salicylic acid in configuring the low-temperature transcriptome and freezing tolerance of *Arabidopsis* . Plant J. 2013;75: 364–376. 10.1111/tpj.12205 23581962

[23] Ding Y, Li H, Zhang X, Xie Q, Gong Z, Yang S. OST1 kinase modulates freezing tolerance by enhancing ICE1 stability in Arabidopsis. Dev Cell. 2015;32: 278–289. 10.1016/j.devcel.2014.12.023 25669882

[24] Peng X, Wu Q, Teng L, Tang F, Pi Z, Shen S. Transcriptional regulation of the paper mulberry under cold stress as revealed by a comprehensive analysis of transcription factors. BMC Plant Biol. 2015;15: 108 10.1186/s12870-015-0489-2 25928853 PMC4432934

[25] Kidokoro S, Watanabe K, Ohori T, Moriwaki T, Maruyama K, Mizoi J, et al Soybean DREB1/CBF-type transcription factors function in heat and drought as well as cold stress-responsive gene expression. Plant J. 2015;81: 505–518. 10.1111/tpj.12746 25495120

[26] Byun MY, Lee J, Cui LH, Kang Y, Oh TK, Park H, et al Constitutive expression of *DaCBF7*, an Antarctic vascular plant *Deschampsia antarctica* CBF homolog, resulted in improved cold tolerance in transgenic rice plants. Plant Sci. 2015;236: 61–74. 10.1016/j.plantsci.2015.03.020 26025521

[27] Feki K, Quintero FJ, Khoudi H, Leidi EO, Masmoudi K, Pardo JM, et al A constitutively active form of a durum wheat Na^+^/H^+^ antiporter SOS1 confers high salt tolerance to transgenic *Arabidopsis* . Plant Cell Rep. 2014;33: 277–88. 10.1007/s00299-013-1528-9 24150094

[28] Lin H, Du W, Yang Y, Schumaker KS, Guo Y. A calcium-independent activation of the *Arabidopsis* SOS2-like protein kinase24 by its interacting SOS3-like calcium binding protein1. Plant Physiol. 2014;164: 2197–2206. 10.1104/pp.113.232272 24521877 PMC3982772

[29] Zhou H, Lin H, Chen S, Becker K, Yang Y, Zhao J, et al Inhibition of the *Arabidopsis* salt overly sensitive pathway by 14-3-3 proteins. Plant Cell. 2014;26: 1166–82. 10.1105/tpc.113.117069 24659330 PMC4001376

[30] Yang Y, Tang RJ, Jiang CM, Li B, Kang T, Liu H, et al Overexpression of the *PtSOS2* gene improves tolerance to salt stress in transgenic poplar plants. Plant Biotechnol J. 2015; 10.1111/pbi.12335 25641517

[31] Sakuma Y, Maruyama K, Osakabe Y, Qin F, Seki M, Shinozaki K, et al Functional analysis of an *Arabidopsis* transcription factor, DREB2A, involved in drought-responsive gene expression. Plant Cell. 2006;18: 1292–1309. 16617101 10.1105/tpc.105.035881 PMC1456870

[32] Wang F, Chen HW, Li QT, Wei W, Li W, Zhang WK, et al GmWRKY27 interacts with GmMYB174 to reduce expression of GmNAC29 for stress tolerance in soybean plants. Plant J. 2015; 10.1111/tpj.12879 25990284

[33] Sakuraba Y, Kim YS, Han SH, Lee BD, Paek NC. The *Arabidopsis* transcription factor NAC016 promotes drought stress responses by repressing *AREB1* transcription through a trifurcate feed-forward regulatory loop involving NAP. Plant Cell. 2015; pii: tpc.15.00222 10.1105/tpc.15.00222 PMC4498208 26059204

[34] Hu H, Wang J, Shi C, Yuan C, Peng C, Yin J, et al A receptor like kinase gene with expressional responsiveness on *Xanthomonas oryzae pv*. *oryzae* is essential for *Xa21*-mediated disease resistance. Rice. 2015;8: 34 10.1186/s12284-014-0034-1 26054238 PMC4883590

[35] Burdiak P, Rusaczonek A, Witon D, Glow D, Karpinski S. Cysteine-rich receptor-like kinase CRK5 as a regulator of growth, development, and ultraviolet radiation responses in *Arabidopsis thaliana* . J Exp Bot. 2015;66: 3325–3337. 10.1093/jxb/erv143 25969551 PMC4449547

[36] de Lorenzo L, Merchan F, Laporte P, Thompson R, Clarke J, Sousa C, et al A novel plant leucine-rich repeat receptor kinase regulates the response of *Medicago truncatula* roots to salt stress. Plant Cell. 2009;21: 668–680. 10.1105/tpc.108.059576 19244136 PMC2660638

[37] Ouyang SQ, Liu YF, Liu P, Lei G, He SJ, Ma B, et al Receptor-like kinase OsSIK1 improves drought and salt stress tolerance in rice (*Oryza sativa*) plants. Plant J. 2010;62: 316–329. 10.1111/j.1365-313X.2010.04146.x 20128882

[38] Zhang X, Yang G, Shi R, Han X, Qi L, Wang R, et al *Arabidopsis* cysteine-rich receptor-like kinase 45 functions in the responses to abscisic acid and abiotic stresses. Plant Physiol Biochem. 2013;67: 189–198. 10.1016/j.plaphy.2013.03.013 23583936

[39] Lee DJ, Chi YT, Kim DM, Choi SH, Lee JY, Choi GW. Ectopic expression of *CaRLK1* enhances hypoxia tolerance with increasing alanine production in *Nicotiana* spp. Plant Mol Biol. 2014;86: 255–270. 10.1007/s11103-014-0227-4 25030225

[40] Yang L, Wu K, Gao P, Liu X, Li G, Wu Z. GsLRPK, a novel cold-activated leucine-rich repeat receptor-like protein kinase from *Glycine soja*, is a positive regulator to cold stress tolerance. Plant Sci. 2014;215–216: 19–28. 10.1016/j.plantsci.2013.10.009 24388511

[41] Yang L, Wu K, Gao P, Liu X, Li G, Wu Z. GsLRPK, a novel cold-activated leucine-rich repeat receptor-like protein kinase from *Glycine soja*, is a positive regulator to cold stress tolerance. Plant Sci. 2014;215: 19–28. 10.1016/j.plantsci.2013.10.009 24388511

[42] Park S, Moon JC, Kim JH, Kim DS, Jang CS. Molecular dissection of the response of a rice leucine-rich repeat receptor-like kinase (LRR-RLK) gene to abiotic stresses. J Plant Physiol. 2014;171: 1645–1653. 10.1016/j.jplph.2014.08.002 25173451

[43] Gao LL, Xue HW. Global analysis of expression profiles of rice receptor-like kinase genes. Mol Plant. 2012;5: 143–153. 10.1093/mp/ssr062 21765177

[44] Shiu SH, Bleecker AB. Plant receptor-like kinase gene family: diversity, function, and signaling. Sci Signal. 2001;2011: re22. 10.1126/stke.2001.113.re22 11752632

[45] Shiu SH, Karlowski WM, Pan R, Tzeng YH, Mayer KF, Li WH. Comparative analysis of the receptor-like kinase family in *Arabidopsis* and rice. Plant Cell. 2004;16: 1220–1234. 15105442 10.1105/tpc.020834 PMC423211

[46] Diray-Arce J, Clement M, Gul B, Khan MA, Nielsen BL. Transcriptome assembly, profiling and differential gene expression analysis of the halophyte *Suaeda fruticosa* provides insights into salt tolerance. BMC Genomics. 2015;16: 353 10.1186/s12864-015-1553-x 25943316 PMC4422317

[47] Yang X, Yu XY, Li YF. *De novo* assembly and characterization of the baryardgrass (*Echinochloa crus-galli*) transcriptome using next-generation pyrosequencing. PLoS One. 2013;8: e69168 10.1371/journal.pone.0069168 23874903 PMC3707877

[48] Ye CY, Lin Z, Li G, Wang YY, Qiu J, Fu F, et al *Echinochloa* chloroplast genomes: Insights into the evolution and taxonomic identification of two weedy species. PLoS One. 2014;9: e113657 10.1371/journal.pone.0113657 25427255 PMC4245208

[49] Livak KJ, Schmittgen TD. Analysis of relative gene expression data using real-time quantitative PCR and the delta delta CT method. Methods. 2001;25: 402–408. 11846609 10.1006/meth.2001.1262

[50] Yabuno T. Biology of *Echinochloa* species. In: Proceedings of the Conference IRRI Weed Control in Rice: Los Banos, Philippines: 1983. pp. 308–311.

[51] Liu S, Li W, Wu Y, Chen C, Lei J. *De novo* transcriptome assembly in chili pepper (*Capsicum frutescens*) to identify genes involved in the biosynthesis of capsaicinoids. Plos One. 2013;8:e48156 10.1371/journal.pone.0048156 23349661 PMC3551913

[52] Lee BH, Henderson DA, Zhu JK. The *Arabidopsis* cold-responsive transcriptome and its regulation by ICE1. Plant Cell. 2005;17: 3155–3175. 16214899 10.1105/tpc.105.035568 PMC1276035

[53] Suzuki N, Rizhsky L, Liang H, Shuman J, Shulaev V, Mittler R. Enhanced tolerance to environmental stress in transgenic plants expressing the transcriptional coactivator multiprotein bridging factor 1c. Plant Physiol. 2005;139: 1313–1322. 16244138 10.1104/pp.105.070110 PMC1283768

[54] Ding Y, Liu N, Virlouvet L, Riethoven JJ, Fromm M, Avramova Z. Four distinct types of dehydration stress memory genes in *Arabidopsis thaliana* . BMC Plant Biol. 2013;13: 229 10.1186/1471-2229-13-229 24377444 PMC3879431

[55] CBOL Plant Workgin Group. A DNA barcode for land plants. Proc Natl Acad Sci U S A. 2009;106: 12794–12797. 10.1073/pnas.0905845106 19666622 PMC2722355

[56] Doust A, Kellogg E, Devos K, Bennetzen J. Foxtail millet: a sequence-driven grass model system. Plant Physiol. 2009; 149:137–141. 10.1104/pp.108.129627 19126705 PMC2613750

[57] Zhang G, Liu X, Quan Z, Cheng S, Xu X, Pan S, et al Genome sequence of foxtail millet (*Setaria italica*) provides insights into grass evolution and biofuel potential. Nature Biotechnol. 2012; 30: 549–554. 22580950 10.1038/nbt.2195

[58] Lata C, Gupta S, Prasad M. Foxtail millet: a model crop for genetic and genomic studies in bioenergy grasses. Crit Rev Biotechnol. 2013; 33: 328–343. 10.3109/07388551.2012.716809 22985089

[59] Osakabe Y, Yamaguchi-Shinozaki K, Shinozaki K, Tran LS. Sensing the environment: key roles of membrane-localized kinases in plant perception and response to abiotic stress. J Exp Bot. 2013;64: 445–458. 10.1093/jxb/ers354 23307915

[60] Sinha AK, Jaggi M, Raghuram B, Tuteja N. Mitogen-activated protein kinase signaling in plants under abiotic stress. Plant Signal Behav. 2011;6: 196–203. 21512321 10.4161/psb.6.2.14701 PMC3121978

[61] Tena G, Boudsocq M, Sheen J. Protein kinase signaling networks in plant innate immunity. Curr Opin Plant Biol. 2011;14: 519–529. 10.1016/j.pbi.2011.05.006 21704551 PMC3191242

[62] Afzal AJ, Wood AJ, Lightfoot DA. Plant receptor-like serine threonine kinases: roles in signaling and plant defense. Mol Plant Microbe Interact. 2008;21: 507–517. 10.1094/MPMI-21-5-0507 18393610

[63] Shiu SH, Bleecker AB. Expansion of the receptor-like kinase/pelle gene family and receptor-like proteins in *Arabidopsis* . Plant Physiol. 2003;132: 530–543. 12805585 10.1104/pp.103.021964 PMC166995

[64] Shiu SH, Bleecker AB. Receptor-like kinases from Arabidopsis form a monophyletic gene family related to animal receptor kinases. Proc Natl Acad Sci U S A. 2001;98: 10763–10768. 11526204 10.1073/pnas.181141598 PMC58549

[65] Flagel LE, Wendel JF. Gene duplication and evolutionary novelty in plants. New Phytol. 2009;183: 557–564. 10.1111/j.1469-8137.2009.02923.x 19555435

[66] Yang T, Chaudhuri S, Yang L, Du L, Poovaiah BW. A calcium/calmodulin-regulated member of the receptor-like kinase family confers cold tolerance in plants. J Biol Chem. 2009; 285: 7119–7126. 20026608 10.1074/jbc.M109.035659 PMC2844161

[67] Osakabe Y, Maruyama K, Seki M, Satou M, Shinozaki K, Yamaguchi-Shinozaki K. Leucine-rich repeat receptor-like kinase1 is a key membrane-bound regulator of abscisic acid early signaling in *Arabidopsis* . Plant Cell. 2005;17: 1105–1119. 15772289 10.1105/tpc.104.027474 PMC1087989

[68] Osakabe Y, Mizuno S, Tanaka H, Maruyama K, Osakabe K, Todaka D, et al Overproduction of the membrane-bound receptor-like protein kinase 1, RPK1, enhances abiotic stress tolerance in Arabidopsis. J Biol Chem. 2010;285: 9190–9201. 10.1074/jbc.M109.051938 20089852 PMC2838338

[69] Tanaka H, Osakabe Y, Katsura S, Mizuno S, Maruyama K, Kusakabe K, et al Abiotic stress-inducible receptor-like kinases negatively control ABA signaling in *Arabidopsis* . Plant J. 2012;70: 599–613. 10.1111/j.1365-313X.2012.04901.x 22225700

[70] Giri J, Vij S, Dansana PK, Tyagi AK. Rice A20/AN1 zinc-finger containing stress-associated proteins (SAP1/11) and a receptor-like cytoplasmic kinase (OsRLCK253) interact via A20 zinc-finger and confer abiotic stress tolerance in transgenic *Arabidopsis* plants. New Phytol. 2011;191: 721–732. 10.1111/j.1469-8137.2011.03740.x 21534973

[71] Gamuyao R, Chin JH, Pariasca-Tanaka J, Pesaresi P, Catausan S, Dalid C, et al The protein kinase Pstol1 from traditional rice confers tolerance of phosphorus deficiency. Nature. 2012;488: 535–539. 10.1038/nature11346 22914168

[72] Yang L, Ji W, Zhu Y, Gao P, Li Y, Cai H, et al GsCBRLK, a calcium/calmodulin-binding receptor-like kinase, is a positive regulator of plant tolerance to salt and ABA stress. J Exp Bot. 2010;61: 2519–2533. 10.1093/jxb/erq084 20400529

[73] Pitorre D, Llauro C, Jobet E, Guilleminot J, Brizard JP, Delseny M, et al RLK7, a leucine-rich repeat receptor-like kinase, is required for proper germination speed and tolerance to oxidative stress in *Arabidopsis thaliana* . Planta. 2010;232: 1339–1353. 10.1007/s00425-010-1260-4 20811905

[74] Chen F, Fasoli M, Tornielli GB, Dal Santo S, Pezzotti M, Zhang L, et al The evolutionary history and diverse physiological roles of the grapevine calcium-dependent protein kinase gene family. PLoS One. 2013;8: e80818 10.1371/journal.pone.0080818 24324631 PMC3855637

[75] Voesenek LA, Bailey-Serres J. Flooding tolerance: O_2_ sensing and survival strategies. Curr Opin Plant Biol. 2013;16: 647–653. 10.1016/j.pbi.2013.06.008 23830867

[76] Xu ZY, Lee KH, Dong T, Jeong JC, Jin JB, Kanno Y. et al A vacuolar β-glucosidase homolog that possesses glucose-conjugated abscisic acid hydrolyzing activity plays an important role in osmotic stress responses in *Arabidopsis* . Plant Cell. 2012;24: 2184–2199. 10.1105/tpc.112.095935 22582100 PMC3442595

[77] Tseng C, Hong C-Y, Yu S-M, and Ho T-H. Abscisic acid- and stress-induced highly proline-rich glycoproteins regulate root growth in rice. Plant Physiology. 2013;163: 118–134. 10.1104/pp.113.217547 23886623 PMC3762635

[78] Le Gall H, Philippe F, Domon J-M, Gillet F, Pelloux J, Rayon C. Cell wall metabolism in response to abiotic stress. Plants. 2015;4: 112–166. 27135320 10.3390/plants4010112 PMC4844334

[79] Blanco-Herrera F, Moreno AA, Tapia R, Reyes F, Araya M, D’Alessio C, et al The UDP-glucose: glycoprotein glucosyltransferase (UGGT), a key enzyme in ER quality control, plays a significant role in plant growth as well as biotic and abiotic stress in *Arabidopsis* thaliana. BMC Plant Biol. 2015;15: 127 10.1186/s12870-015-0525-2 26017403 PMC4465474

[80] von Schaewen A, Rips S, Jeong IS, Koiwa H. *Arabidopsis thaliana* KORRIGAN1 protein: N-glycan modification, localization, and function in cellulose biosynthesis and osmotic stress responses. Plant Signal Behav. 2015;10: e1024397 10.1080/15592324.2015.1024397 26039485 PMC4622505

