# Correction: Uncovering the Differential Molecular Basis of Adaptive Diversity in Three *Echinochloa* Leaf Transcriptomes

**DOI:** 10.1371/journal.pone.0139534

**Published:** 2015-09-25

**Authors:** The PLOS ONE Staff

In the Funding section, the project code from the funder Cooperative Research Program for Agriculture Science & Technology Development is listed incorrectly. The correct project code is: 01126901. The publisher apologizes for the error.
