# Supplementary figures and images for: Uncovering the Differential Molecular Basis of Adaptive Diversity in Three Echinochloa Leaf Transcriptomes
Source: PLoS One. 2015 Aug 12;10(8):e0134419. doi: 10.1371/journal.pone.0134419 (PMC4534374; doi:10.1371/journal.pone.0134419)

**F-type EOZ**

**EC-SNU 1**

**EC-SNU 2**

**EC-SNU 3**

**(A)**

**1mm**

**(B)**

**1mm**

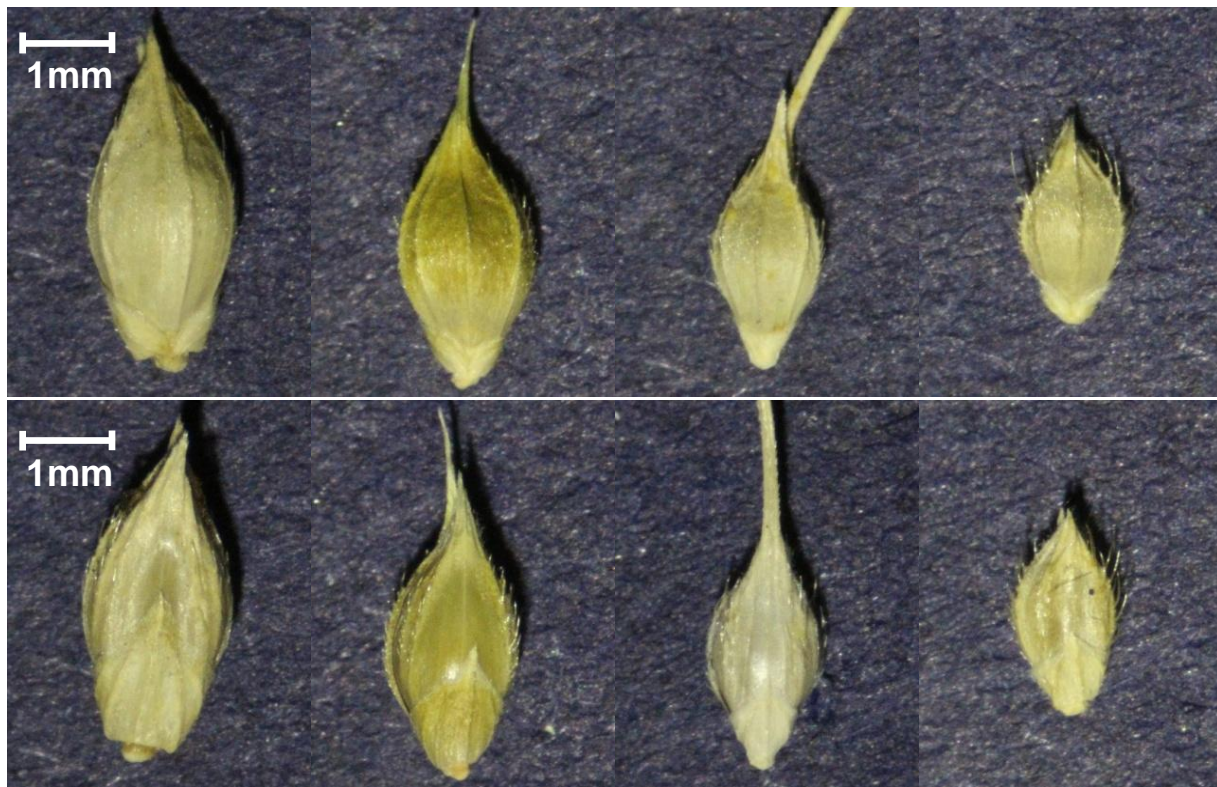

Supplement: S1 Fig — (A) E. oryzicola, (B) EC-SNU1, (C) EC-SNU2, and (D) EC-SNU3. (PDF) [file pone.0134419.s001.pdf]

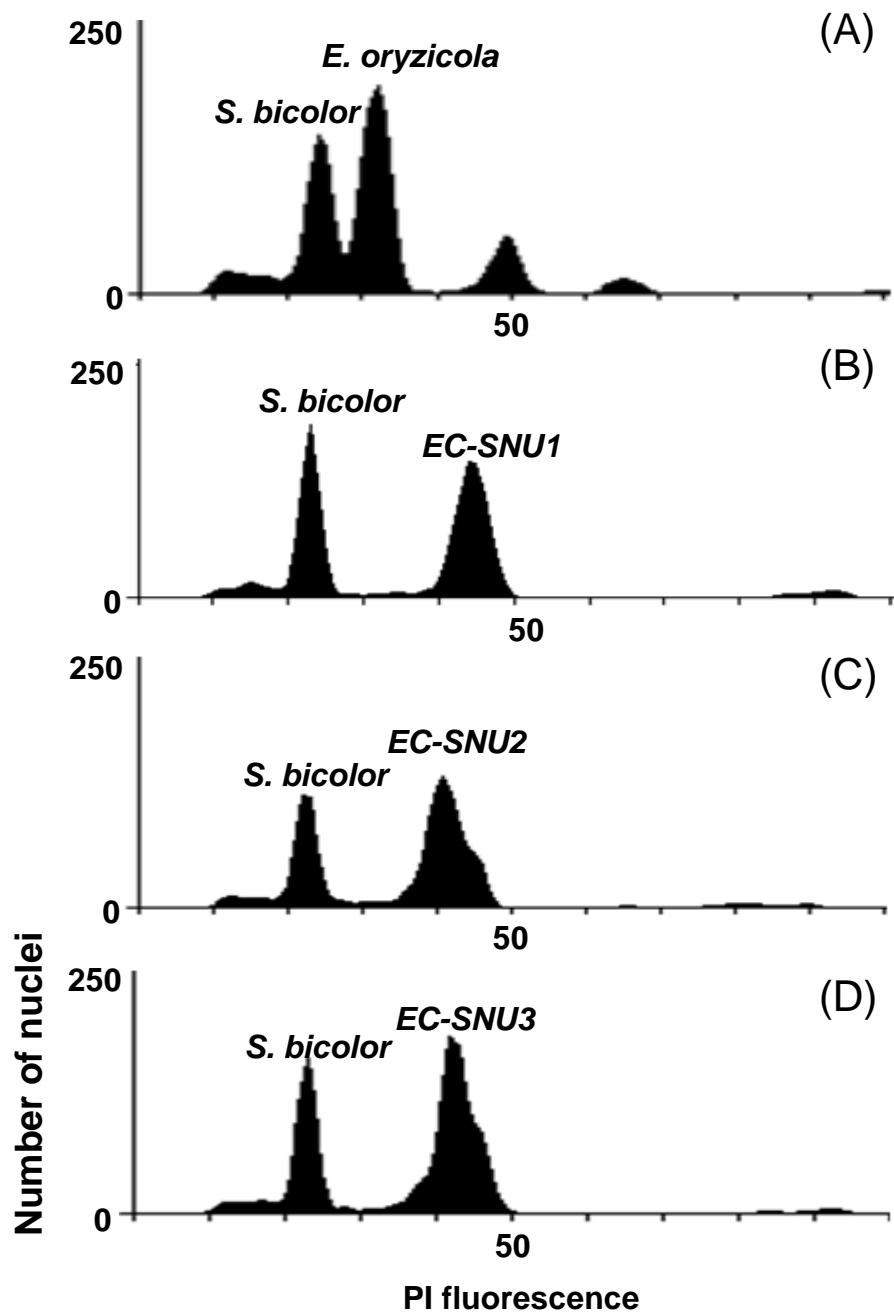

Supplement: S2 Fig — (PDF) [file pone.0134419.s002.pdf]

(A) *E. oryzicola* (4X)

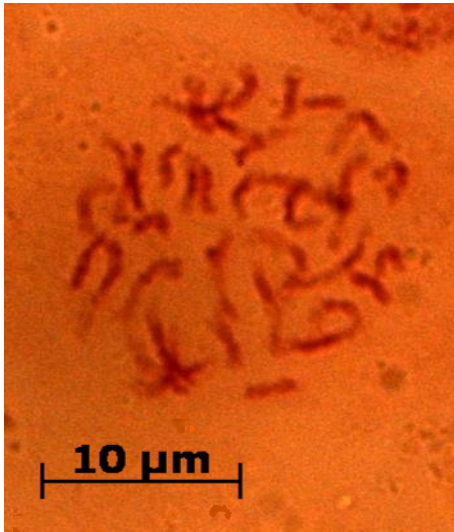

(B) EC-SNU1 (6X)

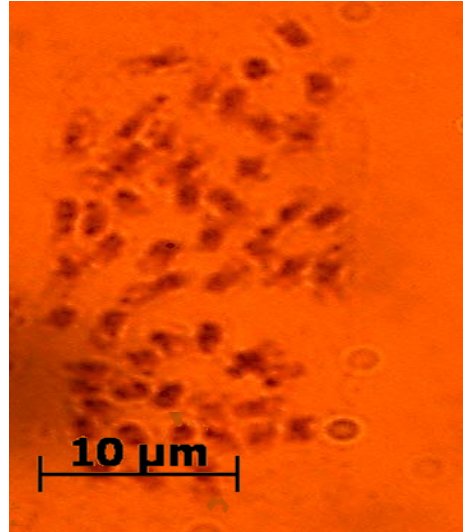

(C) EC-SNU2 (6X)

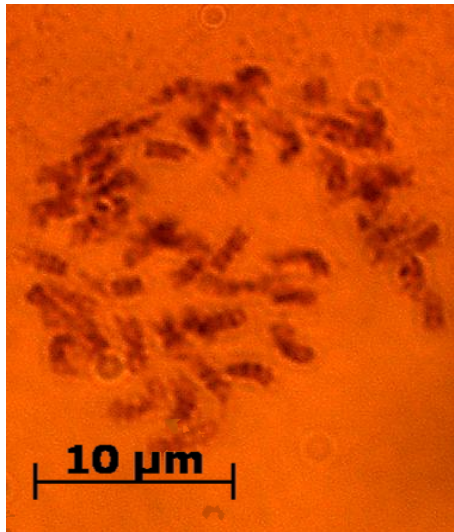

(D) EC-SNU3 (6X)

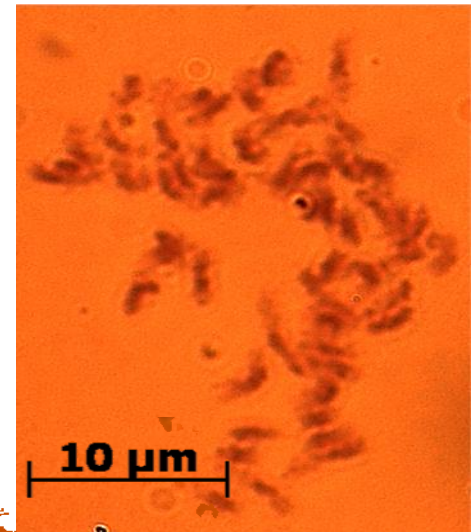

Supplement: S3 Fig — (PDF) [file pone.0134419.s003.pdf]

■ EC-SNU1

■ EC-SNU2

■ EC-SNU3

(A)

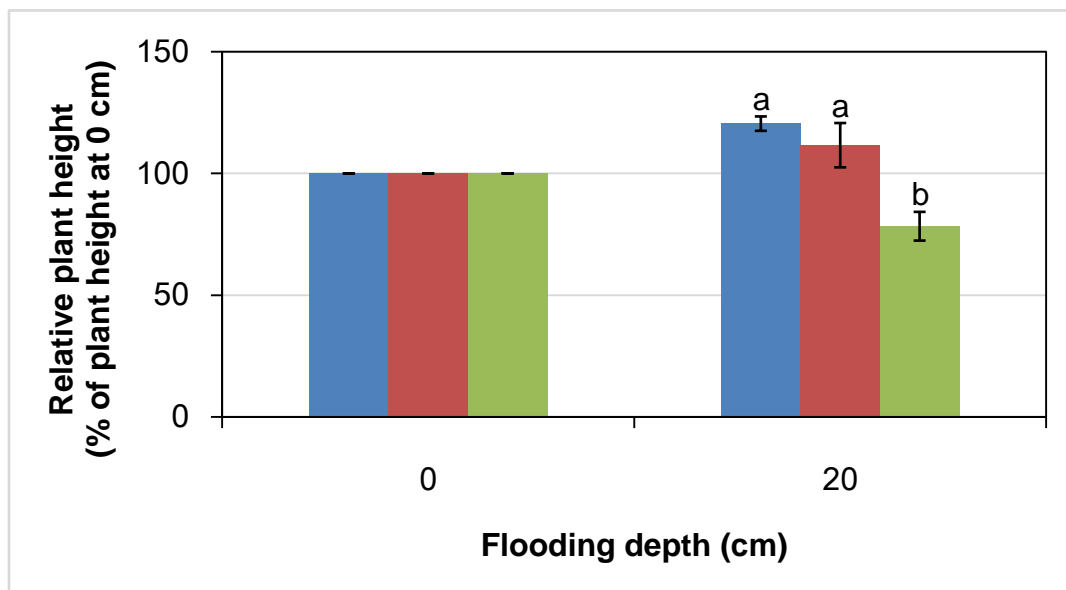

(B)

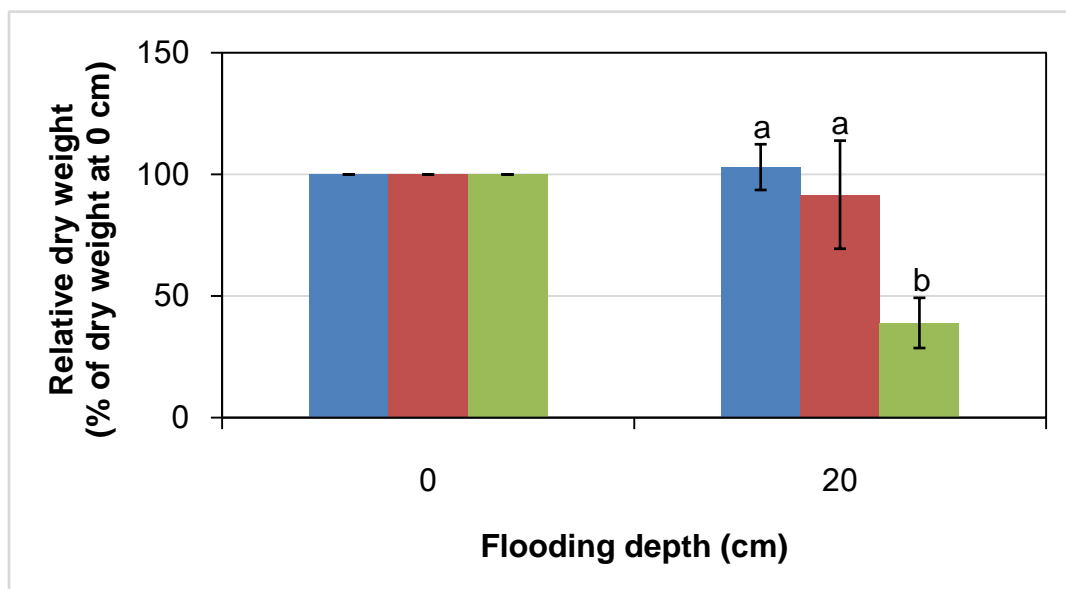

Supplement: S4 Fig — (PDF) [file pone.0134419.s004.pdf]

### Biological Process

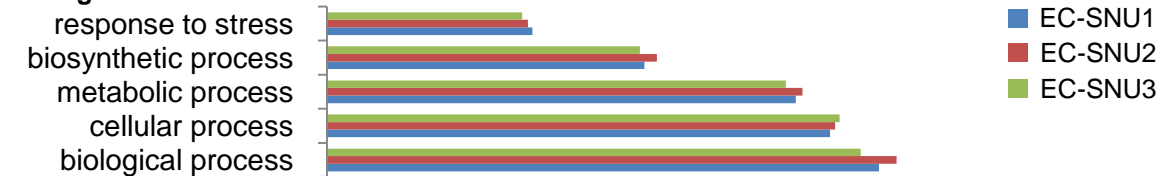

### Cellular Process

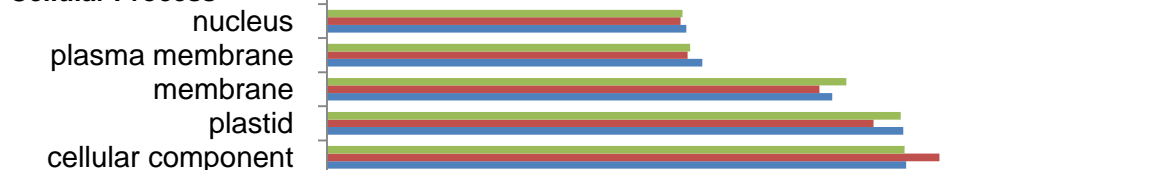

### Molecular Process

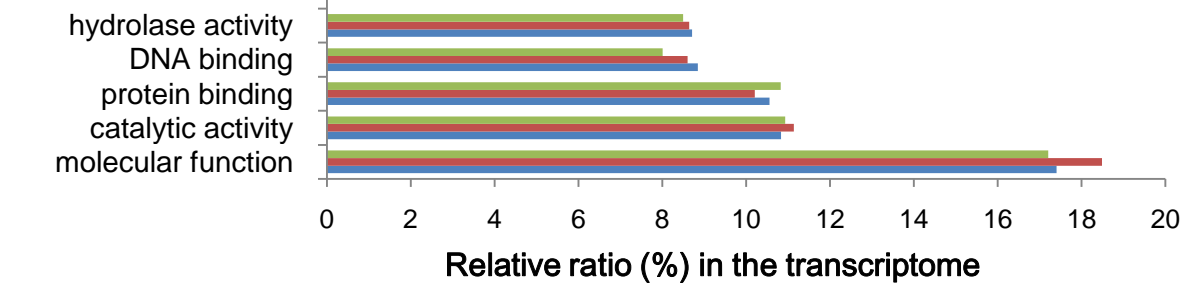

Supplement: S5 Fig — (PDF) [file pone.0134419.s005.pdf]

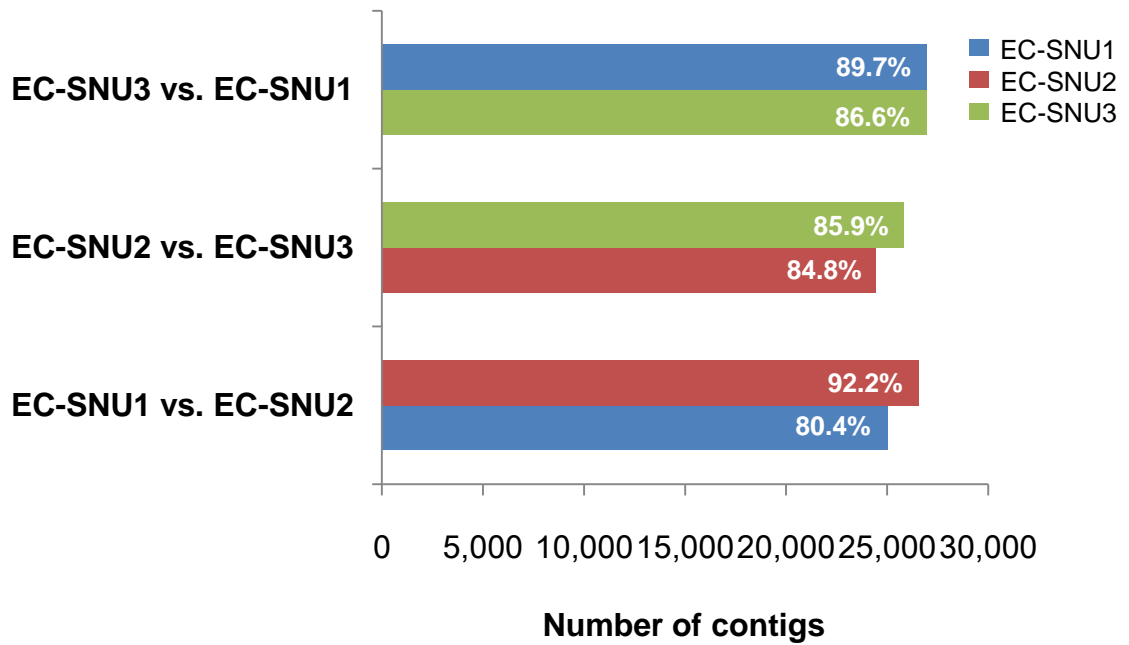

Supplement: S6 Fig — (PDF) [file pone.0134419.s006.pdf]

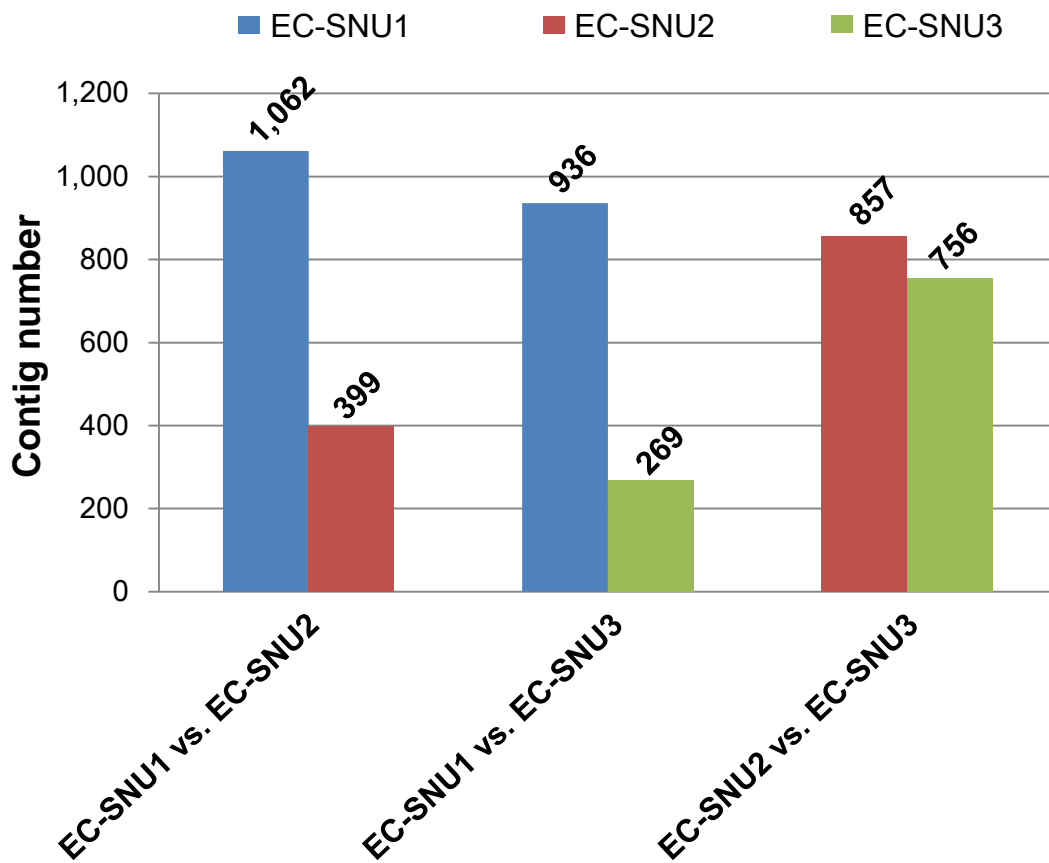

Supplement: S7 Fig — (PDF) [file pone.0134419.s007.pdf]

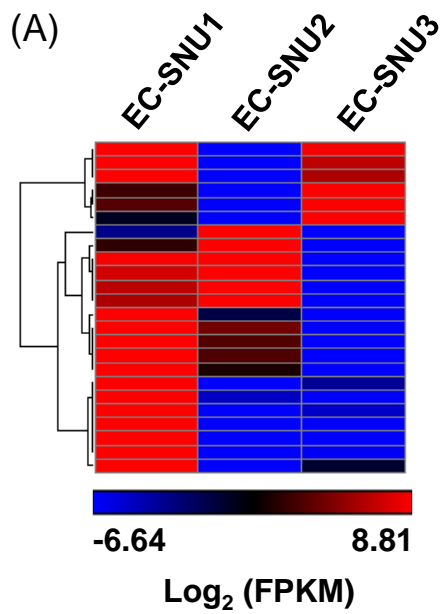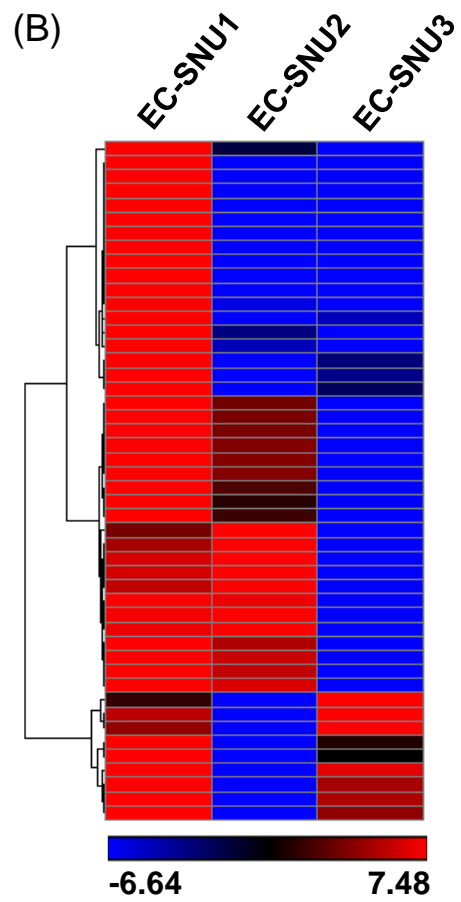

Supplement: S8 Fig — (PDF) [file pone.0134419.s008.pdf]

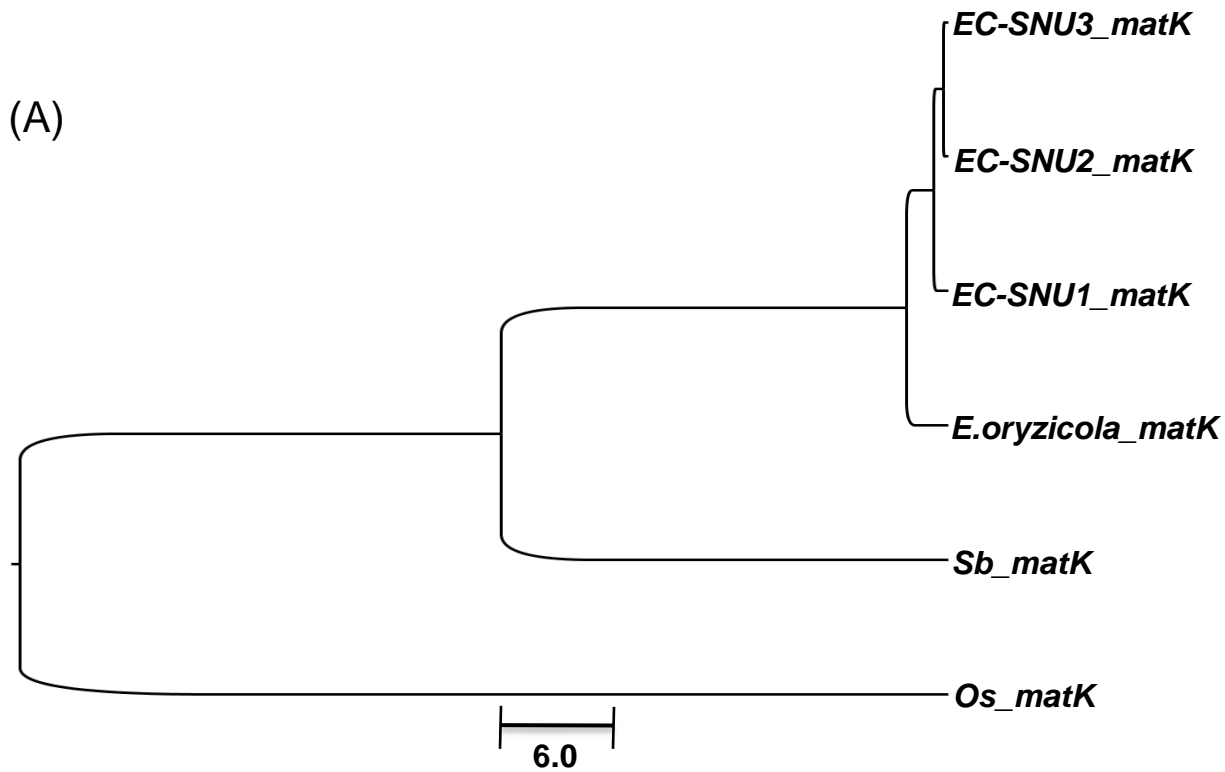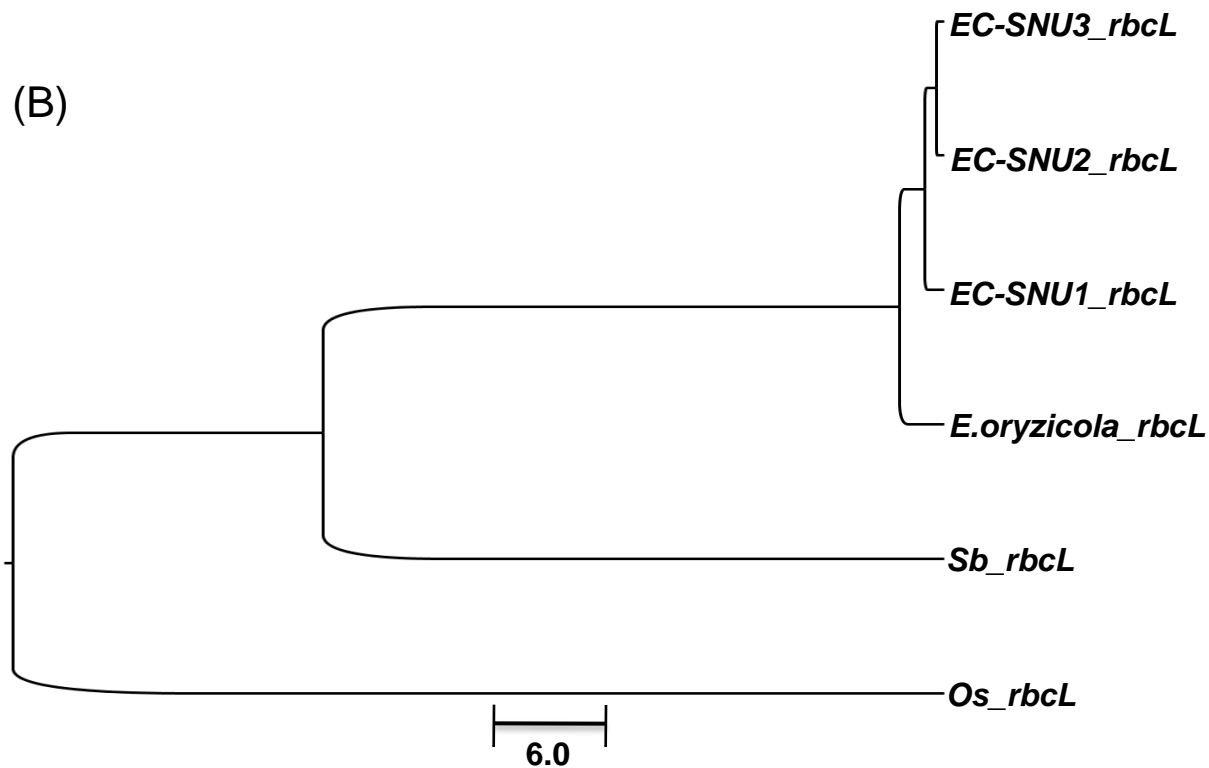

Supplement: S9 Fig — (PDF) [file pone.0134419.s009.pdf]
