# Supplementary material for: Uncovering the Differential Molecular Basis of Adaptive Diversity in Three Echinochloa Leaf Transcriptomes
Source: PLoS One. 2015 Aug 12;10(8):e0134419. doi: 10.1371/journal.pone.0134419 (PMC4534374; doi:10.1371/journal.pone.0134419)
Supplement: S1 Table — (DOCX) [file pone.0134419.s010.docx]

**S1 Table.** Primers used for qRT-PCR validation of selected DEGs.

| **Contig ID** | **Annotation** | **Forward** | **Reverse** |
| --- | --- | --- | --- |
| EC-SNU1_contig_11722 | Cysteine-rich RLK 7 | GGTCTCTCAAGGTGTTTTGATG | GTGCCATATATCCCGGTGTTCC |
| EC-SNU1_contig_12868 | LRR family | TGGACCTCTCCGGGAACAAGA | GATGAGGTTGCCGGAGAGGTC |
| EC-SNU1_contig_13763 | SOS3-interacting protein 1 | GAACTCCAGCTTATGTTGCTC | TCCACAAGACCATATGTCAGC |
| EC-SNU1_contig_15644 | Cysteine-rich RLK 25 | GGCAGCCCGATGGTGAAGCTGC | CAGTGGCCGGCACAACATCCGT |
| EC-SNU1_contig_18930 | NB-ARC domain protein | ATTGCTGACCTCAAGATCAGGG | TGCCATTGCTGTTGCTTGGGTT |
| EC-SNU1_contig_20550 | ERF domain protein 9 | CGGCCAGTTCAGATCCAGATCG | GTCGGTGGTCGATCACACCCCT |
| EC-SNU1_contig_2280 | CDPK 6 | TCCGTATCGACTTCAGCAATAA | TGATGAGTTGGAAGAAGCTCTG |
| EC-SNU1_contig_24131 | CDPK 1 | GATCTGGATCTCGCGGCGCACG | GGAAGCAGTTCGCCTGCAAGTC |
| EC-SNU1_contig_24723 | CDPK family | CCCTCATGCTGATATTCAGGCT | TATGGAGAGTTTGTTGCCATGA |
| EC-SNU1_contig_2476 | Cysteine-rich RLK 40 | CTCGTGAAGTTAGAGCATGCAT | TCAGTTTATCTACAATCTCGG |
| EC-SNU1_contig_29736 | Calmodulin 1 | AAGAACTGGGAGCTGTGATGA | TCCATTTCCATCAGCATCAAC |
| EC-SNU1_contig_30042 | Cysteine-rich RLK 39 | GTATACATGCCACCAGAATACA | GTATAATTATAACACCCAAACTG |
| EC-SNU1_contig_30729 | SOS3-interacting protein 1 | CATGGTGAAGAAACAGGACGG | GGTGTCACCTCGAAGATCTC |
| EC-SNU1_contig_31089 | Cysteine-rich RLK 6 | GAATATGCGGTTCTTGGACATG | TCTTCCTGTCACAATTTCCAGG |
| EC-SNU1_contig_3146 | NB-ARC domain protein | GCTCAAGAGAGGATGATAACCA | ACCCTCAAATGTTCCAGTGCTT |
| EC-SNU1_contig_4484 | LRR-related | CTTCTGGTGTAAGTGCATGAGA | ACTTCTCCTCAGTTTAGGCCAG |
| EC-SNU1_contig_5514 | NB-ARC domain protein | AGAAGCATGATCCAACCTGTTG | TGATGTAATCAAGAATGATGTC |
| EC-SNU1_contig_6365 | Cysteine-rich RLK29 | GTCAGGAATGGCCACTGACGAC | CCTTCAGAAACTCTGCTGGTAGT |
| EC-SNU1_contig_9222 | NB-ARC domain protein | ACCATTGAAGAGTGGGACCAGA | ATAAGCTTGCAACTTCAACTTG |
