# Supplementary material for: Uncovering the Differential Molecular Basis of Adaptive Diversity in Three Echinochloa Leaf Transcriptomes
Source: PLoS One. 2015 Aug 12;10(8):e0134419. doi: 10.1371/journal.pone.0134419 (PMC4534374; doi:10.1371/journal.pone.0134419)
Supplement: S2 Table — (DOCX) [file pone.0134419.s011.docx]

**S2 Table.** GO categorization in molecular process of three *E. crus-galli* transcriptomes.

| **Molecular function** | **GO id** | **EC-SNU1** |  | **EC-SNU2** |  | **EC-SNU3** |  |
| --- | --- | --- | --- | --- | --- | --- | --- |
| **GO term** |  | **No. of contigs** | **Percent** | **No. of contigs** | **Percent** | **No. of contigs** | **Percent** |
| Binding | GO:0005488 | 931 | 5.45 | 1014 | 5.94 | 1010 | 6.00 |
| Carbohydrate binding | GO:0030246 | 89 | 0.52 | 100 | 0.59 | 90 | 0.54 |
| Catalytic activity | GO:0003824 | 1849 | 10.83 | 1901 | 11.14 | 1839 | 10.93 |
| Chromatin binding | GO:0003682 | 32 | 0.19 | 26 | 0.15 | 35 | 0.21 |
| DNA binding | GO:0003677 | 1510 | 8.85 | 1468 | 8.60 | 1347 | 8.01 |
| Enzyme regulator activity | GO:0030234 | 119 | 0.70 | 116 | 0.68 | 115 | 0.68 |
| Hydrolase activity | GO:0016787 | 1487 | 8.71 | 1475 | 8.64 | 1429 | 8.5 |
| Kinase activity | GO:0016301 | 813 | 4.76 | 730 | 4.28 | 788 | 4.68 |
| Lipid binding | GO:0006630 | 131 | 0.77 | 109 | 0.64 | 123 | 0.73 |
| Molecular_function | GO:0008369 | 2971 | 17.41 | 3155 | 18.48 | 2894 | 17.21 |
| Motor activity | GO:0003774 | 47 | 0.28 | 38 | 0.22 | 36 | 0.21 |
| Nuclease activity | GO:0004518 | 113 | 0.66 | 120 | 0.70 | 106 | 0.63 |
| Nucleic acid binding | GO:0003676 | 414 | 2.43 | 475 | 2.78 | 460 | 2.73 |
| Nucleotide binding | GO:0000166 | 1300 | 7.62 | 1112 | 6.52 | 1221 | 7.26 |
| Oxygen binding | GO:0019825 | 69 | 0.40 | 79 | 0.46 | 78 | 0.46 |
| Protein binding | GO:0005515 | 1802 | 10.56 | 1742 | 10.21 | 1821 | 10.83 |
| Receptor activity | GO:0004872 | 76 | 0.45 | 53 | 0.31 | 69 | 0.41 |
| Receptor binding | GO:0005102 | 29 | 0.17 | 25 | 0.15 | 32 | 0.19 |
| RNA binding | GO:0003723 | 367 | 2.15 | 376 | 2.20 | 400 | 2.38 |
| DNA binding transcription factor activity | GO:0003700 | 748 | 4.38 | 700 | 4.10 | 646 | 3.84 |
| Signal transducer activity | GO:0004871 | 141 | 0.83 | 87 | 0.51 | 118 | 0.7 |
| Structural molecule activity | GO:0005198 | 329 | 1.93 | 365 | 2.14 | 365 | 2.17 |
| Transferase activity | GO:0016740 | 901 | 5.28 | 987 | 5.78 | 934 | 5.55 |
| Translation factor activity, nucleic acid binding | GO:0008135 | 75 | 0.44 | 95 | 0.56 | 87 | 0.52 |
| Translation regulator activity | GO:0045182 | 6 | 0.04 | 4 | 0.02 | 7 | 0.04 |
| Transporter activity | GO:0005215 | 719 | 4.21 | 716 | 4.19 | 770 | 4.58 |
