# Supplementary material for: Uncovering the Differential Molecular Basis of Adaptive Diversity in Three Echinochloa Leaf Transcriptomes
Source: PLoS One. 2015 Aug 12;10(8):e0134419. doi: 10.1371/journal.pone.0134419 (PMC4534374; doi:10.1371/journal.pone.0134419)
Supplement: S3 Table — (DOCX) [file pone.0134419.s012.docx]

**S3 Table.** GO categorization in cellular process of three *E. crus-galli* transcriptomes.

| **Cellular function** | **GO id** | **EC-SNU1** |  | **EC-SNU2** |  | **EC-SNU3** |  |
| --- | --- | --- | --- | --- | --- | --- | --- |
| **GO term** |  | **No. of contigs** | **Percent** | **No. of contigs** | **Percent** | **No. of contigs** | **Percent** |
| Cell | GO:0005623 | 758 | 4.56 | 746 | 4.48 | 684 | 4.04 |
| Cellular_component | GO:0008370 | 2295 | 13.82 | 2434 | 14.61 | 2331 | 13.78 |
| Cell wall | GO:0005618 | 279 | 1.68 | 295 | 1.77 | 302 | 1.79 |
| Cytoplasm | GO:0005737 | 604 | 3.64 | 594 | 3.57 | 637 | 3.77 |
| Cytoskeleton | GO:0005856 | 132 | 0.79 | 115 | 0.69 | 136 | 0.80 |
| Cytosol | GO:0005829 | 1437 | 8.65 | 1517 | 9.11 | 1412 | 8.35 |
| Endoplasmic reticulum | GO:0005783 | 282 | 1.70 | 285 | 1.71 | 299 | 1.77 |
| Endosome | GO:0005768 | 71 | 0.43 | 66 | 0.40 | 60 | 0.35 |
| External encapsulating structure | GO:0030312 | 2 | 0.01 | 1 | 0.01 | 1 | 0.01 |
| Extracellular region | GO:0005576 | 168 | 1.01 | 186 | 1.12 | 174 | 1.03 |
| Extracellular space | GO:0005576 | 3 | 0.02 | 3 | 0.02 | 4 | 0.02 |
| Golgi apparatus | GO:0005794 | 209 | 1.26 | 204 | 1.22 | 218 | 1.29 |
| Intracellular | GO:0005622 | 551 | 3.32 | 572 | 3.43 | 603 | 3.56 |
| Lysosome | GO:0005764 | 1 | 0.01 | 0 | 0.00 | 1 | 0.01 |
| Membrane | GO:0016020 | 2002 | 12.05 | 1957 | 11.75 | 2096 | 12.39 |
| Mitochondrion | GO:0005739 | 662 | 3.99 | 719 | 4.32 | 716 | 4.23 |
| Nuclear envelope | GO:0005635 | 69 | 0.42 | 60 | 0.36 | 51 | 0.30 |
| Nucleolus | GO:0005730 | 248 | 1.49 | 234 | 1.40 | 255 | 1.51 |
| Nucleoplasm | GO:0005654 | 166 | 1.00 | 147 | 0.88 | 155 | 0.92 |
| Nucleus | GO:0005634 | 1424 | 8.57 | 1405 | 8.44 | 1435 | 8.48 |
| Peroxisome | GO:0005777 | 136 | 0.82 | 137 | 0.82 | 150 | 0.89 |
| Plasma membrane | GO:0005886 | 1487 | 8.95 | 1433 | 8.60 | 1466 | 8.67 |
| Plastid | GO:0009536 | 2283 | 13.75 | 2172 | 13.04 | 2315 | 13.69 |
| Ribosome | GO:0005840 | 278 | 1.67 | 324 | 1.95 | 309 | 1.83 |
| Thylakoid | GO:0009579 | 413 | 2.49 | 400 | 2.40 | 461 | 2.73 |
| Vacuole | GO:0005773 | 649 | 3.91 | 650 | 3.90 | 644 | 3.81 |
