# Supplementary material for: Uncovering the Differential Molecular Basis of Adaptive Diversity in Three Echinochloa Leaf Transcriptomes
Source: PLoS One. 2015 Aug 12;10(8):e0134419. doi: 10.1371/journal.pone.0134419 (PMC4534374; doi:10.1371/journal.pone.0134419)
Supplement: S4 Table — (DOCX) [file pone.0134419.s013.docx]

**S4 Table.** GO categorization in biological process of three *E. crus-galli* transcriptomes.

| **Biological function** | **GO id** | **EC-SNU1** |  | **EC-SNU2** |  | **EC-SNU3** |  |
| --- | --- | --- | --- | --- | --- | --- | --- |
| **GO term** |  | **No. of contigs** | **Percent** | **No. of contigs** | **Percent** | **No. of contigs** | **Percent** |
| Abscission | GO:0009838 | 10 | 0.03 | 4 | 0.01 | 8 | 0.03 |
| Anatomical structure morphogenesis | GO:0009653 | 442 | 1.43 | 363 | 1.17 | 419 | 1.35 |
| Behavior | GO:0007610 | 2 | 0.01 | 3 | 0.01 | 1 | 0.00 |
| Biological_process | GO:0008371 | 4084 | 13.17 | 4203 | 13.59 | 3942 | 12.74 |
| Biosynthetic process | GO:0009058 | 2348 | 7.57 | 2435 | 7.87 | 2311 | 7.47 |
| Carbohydrate metabolic process | GO:0005975 | 469 | 1.51 | 513 | 1.66 | 461 | 1.49 |
| Catabolic process | GO:0009056 | 787 | 2.54 | 803 | 2.60 | 837 | 2.70 |
| Cell-cell signaling | GO:0007267 | 23 | 0.07 | 13 | 0.04 | 15 | 0.05 |
| Cell communication | GO:0007154 | 78 | 0.25 | 108 | 0.35 | 83 | 0.27 |
| Cell cycle | GO:0007049 | 202 | 0.65 | 189 | 0.61 | 174 | 0.56 |
| Cell death | GO:0008219 | 147 | 0.47 | 127 | 0.41 | 103 | 0.33 |
| Cell differentiation | GO:0030154 | 315 | 1.02 | 261 | 0.84 | 307 | 0.99 |
| Cell growth | GO:0016049 | 190 | 0.61 | 178 | 0.58 | 186 | 0.60 |
| Cellular component organization | GO:0016043 | 775 | 2.50 | 777 | 2.51 | 828 | 2.68 |
| Cellular homeostasis | GO:0019725 | 131 | 0.42 | 119 | 0.38 | 146 | 0.47 |
| Cellular process | GO:0009987 | 3721 | 12.00 | 3750 | 12.12 | 3785 | 12.23 |
| DNA metabolic process | GO:0006259 | 253 | 0.82 | 234 | 0.76 | 233 | 0.75 |
| Embryo development | GO:0009790 | 293 | 0.94 | 300 | 0.97 | 346 | 1.12 |
| Flower development | GO:0009908 | 210 | 0.68 | 196 | 0.63 | 199 | 0.64 |
| Precursor metabolites and energy | GO:0006091 | 168 | 0.54 | 195 | 0.63 | 208 | 0.67 |
| Growth | GO:0040007 | 62 | 0.20 | 62 | 0.20 | 55 | 0.18 |
| Lipid metabolic process | GO:0006629 | 470 | 1.52 | 530 | 1.71 | 482 | 1.56 |
| Metabolic process | GO:0008152 | 3468 | 11.18 | 3508 | 11.34 | 3389 | 10.95 |
| Multicellular organismal development | GO:0007275 | 784 | 2.53 | 684 | 2.21 | 734 | 2.37 |
| Nucleic acid metabolic process | GO:0090304 | 1877 | 6.05 | 1872 | 6.05 | 1884 | 6.09 |
| Photosynthesis | GO:0015979 | 139 | 0.45 | 151 | 0.49 | 167 | 0.54 |
| Pollen-pistil interaction | GO:0009875 | 35 | 0.11 | 45 | 0.15 | 32 | 0.10 |
| Pollination | GO:0009856 | 62 | 0.20 | 69 | 0.22 | 62 | 0.20 |
| Post-embryonic development | GO:0009791 | 549 | 1.77 | 572 | 1.85 | 657 | 2.12 |
| Protein metabolic process | GO:0019538 | 609 | 1.96 | 599 | 1.94 | 602 | 1.95 |
| Protein modification process | GO:0036211 | 1338 | 4.32 | 1281 | 4.14 | 1325 | 4.28 |
| Epigenetic regulation | GO:0040029 | 99 | 0.32 | 87 | 0.28 | 106 | 0.34 |
| Reproduction | GO:0000003 | 426 | 1.37 | 442 | 1.43 | 525 | 1.70 |
| Response to abiotic stimulus | GO:0009628 | 1010 | 3.26 | 1033 | 3.34 | 1033 | 3.34 |
| Response to biotic stimulus | GO:0009607 | 475 | 1.53 | 466 | 1.51 | 447 | 1.44 |
| Response to endogenous stimulus | GO:0009719 | 694 | 2.24 | 681 | 2.20 | 675 | 2.18 |
| Response to external stimulus | GO:0009605 | 55 | 0.18 | 61 | 0.20 | 53 | 0.17 |
| Response to extracellular stimulus | GO:0009991 | 81 | 0.26 | 99 | 0.32 | 86 | 0.28 |
| Response to stress | GO:0006950 | 1519 | 4.90 | 1483 | 4.79 | 1442 | 4.66 |
| Ripening | GO:0009835 | 0 | 0.00 | 2 | 0.01 | 0 | 0.00 |
| Secondary metabolic process | GO:0019748 | 216 | 0.70 | 184 | 0.59 | 175 | 0.57 |
| Signal transduction | GO:0007165 | 718 | 2.32 | 582 | 1.88 | 682 | 2.20 |
| Translation | GO:0007165 | 410 | 1.32 | 454 | 1.47 | 447 | 1.44 |
| Transport | GO:0006810 | 1214 | 3.92 | 1188 | 3.84 | 1260 | 4.07 |
| Tropism | GO:0009606 | 49 | 0.16 | 27 | 0.09 | 39 | 0.13 |
