# Supplementary material for: Uncovering the Differential Molecular Basis of Adaptive Diversity in Three Echinochloa Leaf Transcriptomes
Source: PLoS One. 2015 Aug 12;10(8):e0134419. doi: 10.1371/journal.pone.0134419 (PMC4534374; doi:10.1371/journal.pone.0134419)
Supplement: S5 Table — (DOCX) [file pone.0134419.s014.docx]

**S5 Table.** Hierarchical clustering of DEGs belonging to serine/threonine protein kinases (STKs).

| **Contig ID** | ***S. bicolor* homolog ID** | ***O. sativa* homolog ID** | **Annotation** |
| --- | --- | --- | --- |
| EC-SNU1_contig_2312 | Sobic.010G020600.1 | LOC_Os06g03970.1 | Receptor-like protein kinase 5 precursor |
| EC-SNU1_contig_2417 | Sobic.004G032900.1 | LOC_Os02g04240.1 | Wee1-like protein kinase |
| EC-SNU1_contig_2418 | Sobic.004G032900.1 | LOC_Os02g04240.1 | Wee1-like protein kinase |
| EC-SNU1_contig_2476 | Sobic.005G096400.1 | LOC_Os11g11890.1 | Protein kinase domain containing protein |
| EC-SNU1_contig_2620 | Sobic.004G214900.1 | LOC_Os02g40240.1 | Receptor kinase |
| EC-SNU1_contig_2679 | Sobic.004G214900.1 | LOC_Os02g40240.1 | Receptor kinase |
| EC-SNU1_contig_3361 | Sobic.006G215400.1 | LOC_Os04g52590.1 | Protein kinase domain containing protein |
| EC-SNU1_contig_3477 | Sobic.003G067600.1 | LOC_Os01g05960.1 | Receptor kinase |
| EC-SNU1_contig_4292 | Sobic.005G075700.1 | LOC_Os12g26940.1 | CHASE domain containing protein |
| EC-SNU1_contig_4484 | Sobic.002G077400.1 | LOC_Os10g33040.1 | Receptor-like protein kinase precursor |
| EC-SNU1_contig_4551 | N/A | N/A | Unknown |
| EC-SNU1_contig_4552 | Sobic.004G214900.1 | LOC_Os02g40240.1 | Receptor kinase |
| EC-SNU1_contig_4657 | Sobic.007G144600.1 | LOC_Os08g34640.1 | Receptor-like protein kinase precursor |
| EC-SNU1_contig_4977 | Sobic.001G530600.1 | LOC_Os03g02680.2 | Cyclin-dependent kinase A-1 |
| EC-SNU1_contig_226 | Sobic.008G022300.1 | LOC_Os11g35120.1 | OsWAK receptor-like cytoplasmic kinase |
| EC-SNU1_contig_5781 | Sobic.003G025300.1 | LOC_Os04g23760.1 | Lectin protein kinase family protein |
| EC-SNU1_contig_6223 | Sobic.002G013800.3 | LOC_Os02g18080.1 | NB-ARC domain containing protein |
| EC-SNU1_contig_6224 | Sobic.002G013800.3 | LOC_Os02g18080.1 | NB-ARC domain containing protein |
| EC-SNU1_contig_6365 | N/A | N/A | Unknown |
| EC-SNU1_contig_7518 | Sobic.004G219700.1 | LOC_Os02g41500.1 | OsWAK13 receptor-like protein kinase |
| EC-SNU1_contig_7541 | Sobic.002G177800.1 | LOC_Os09g18360.1 | protein |
| EC-SNU1_contig_7543 | Sobic.002G177800.1 | LOC_Os09g18360.1 | protein |
| EC-SNU1_contig_7545 | Sobic.002G177800.1 | LOC_Os09g18360.1 | protein |
| EC-SNU1_contig_7934 | Sobic.010G211800.1 | LOC_Os06g22810.1 | CrRLK1L homolog |
| EC-SNU1_contig_8397 | N/A | N/A | Unknown |
| EC-SNU1_contig_8818 | Sobic.002G377400.1 | LOC_Os02g06160.1 | Cysteine-rich receptor-like protein kinase 16 precursor |
| EC-SNU1_contig_8910 | Sobic.004G217500.1 | LOC_Os02g40180.1 | Receptor-like protein kinase 5 precursor |
| EC-SNU1_contig_9958 | Sobic.001G097200.2 | LOC_Os03g52794.1 | Phosphatidylinositol kinase |
| EC-SNU1_contig_11722 | Sobic.005G063600.1 | LOC_Os11g11890.1 | Protein kinase domain containing protein |
| EC-SNU1_contig_11723 | Sobic.005G063600.3 | LOC_Os10g18760.1 | Dirigent |
| EC-SNU1_contig_11726 | Sobic.005G063600.3 | LOC_Os10g18760.1 | Dirigent |
| EC-SNU1_contig_11727 | Sobic.005G063600.3 | LOC_Os10g18760.1 | Dirigent |
| EC-SNU1_contig_12868 | Sobic.003G277900.1 | LOC_Os01g52050.1 | Systemin receptor SR160 precursor |
| EC-SNU1_contig_13598 | Sobic.003G096500.1 | LOC_Os01g02390.1 | Protein kinase domain containing protein |
| EC-SNU1_contig_13763 | N/A | N/A | Unknown |
| EC-SNU1_contig_15397 | N/A | N/A | Unknown |
| EC-SNU1_contig_15643 | Sobic.002G329100.1 | LOC_Os07g35750.1 | TKL_IRAK_DUF26-ld.3 - DUF26 kinases |
| EC-SNU1_contig_15644 | N/A | N/A | Unknown |
| EC-SNU1_contig_16201 | Sobic.001G071700.1 | LOC_Os03g56470.1 | Protein kinase family protein |
| EC-SNU1_contig_17727 | Sobic.001G313100.1 | LOC_Os10g39420.1 | Calcium/calmodulin depedent protein kinases |
| EC-SNU1_contig_17731 | Sobic.001G313100.1 | LOC_Os10g39420.1 | Calcium/calmodulin depedent protein kinases |
| EC-SNU1_contig_18172 | Sobic.007G186100.1 | LOC_Os09g33860.1 | Protein kinase |
| EC-SNU1_contig_19193 | Sobic.009G004900.2 | LOC_Os11g17380.1 | Protein kinase domain containing protein |
| EC-SNU1_contig_19749 | Sobic.001G410100.1 | LOC_Os03g17700.1 | CGMC_MAPKCGMC_2_ERK.2 - CLKC kinases |
| EC-SNU1_contig_19751 | N/A | N/A | Unknown |
| EC-SNU1_contig_22813 | Sobic.003G225200.1 | LOC_Os01g43410.1 | CAMK_like.9 - calcium/calmodulin depedent protein kinases |
| EC-SNU1_contig_24035 | Sobic.002G390100.1 | LOC_Os07g44290.1 | CAMK_like.29 - calcium/calmodulin depedent protein kinases |
| EC-SNU1_contig_24038 | Sobic.003G139500.1 | LOC_Os01g18800.1 | CAMK_like.9 - calcium/calmodulin depedent protein kinases |
| EC-SNU1_contig_24131 | Sobic.002G040800.1 | LOC_Os07g06740.1 | CAMK_like.31 - calcium/calmodulin depedent protein kinases |
| EC-SNU1_contig_24310 | Sobic.004G008100.1 | LOC_Os02g02120.1 | OsWAK11 - OsWAK receptor-like protein kinase |
| EC-SNU1_contig_24296 | Sobic.004G008100.1 | LOC_Os02g02120.1 | OsWAK11 - OsWAK receptor-like protein kinase |
| EC-SNU1_contig_24727 | Sobic.006G192500.1 | LOC_Os04g49510.1 | CAMK_like.27 - calcium/calmodulin depedent protein kinases |
| EC-SNU1_contig_24720 | Sobic.006G192500.1 | LOC_Os04g49510.1 | CAMK_like.27 - calcium/calmodulin depedent protein kinases |
| EC-SNU1_contig_24722 | Sobic.006G192500.1 | LOC_Os04g49510.1 | CAMK_like.27 - calcium/calmodulin depedent protein kinases |
| EC-SNU1_contig_24723 | Sobic.006G192500.1 | LOC_Os04g49510.1 | CAMK_like.27 - calcium/calmodulin depedent protein kinases |
| EC-SNU1_contig_24724 | Sobic.006G192500.1 | LOC_Os04g49510.1 | CAMK_like.27 - calcium/calmodulin depedent protein kinases |
| EC-SNU1_contig_25090 | Sobic.001G390200.1 | LOC_Os03g20380.1 | CAMK_like.2 - calcium/calmodulin depedent protein kinases |
| EC-SNU1_contig_25091 | Sobic.001G390200.1 | LOC_Os03g20380.1 | CAMK_like.2 - calcium/calmodulin depedent protein kinases |
| EC-SNU1_contig_25094 | Sobic.001G390200.1 | LOC_Os03g20380.1 | CAMK_like.2 - calcium/calmodulin depedent protein kinases |
| EC-SNU1_contig_25082 | Sobic.001G390200.1 | LOC_Os03g20380.1 | CAMK_like.2 - calcium/calmodulin depedent protein kinases |
| EC-SNU1_contig_25218 | Sobic.002G290400.1 | LOC_Os09g37949.1 | Serine/threonine-protein kinase SRPK1 |
| EC-SNU1_contig_25220 | Sobic.001G090100.1 | LOC_Os03g53720.1 | SRPK4 |
| EC-SNU1_contig_25540 | N/A | N/A | Unknown |
| EC-SNU1_contig_25525 | Sobic.004G202500.2 | LOC_Os12g27520.1 | Serine/threonine-protein kinase AFC2 |
| EC-SNU1_contig_28077 | Sobic.002G289300.1 | LOC_Os09g37800.1 | Serine/threonine kinase |
| EC-SNU1_contig_28618 | Sobic.005G012200.2 | LOC_Os07g08750.1 | STE_PAK_Ste20_Slob_Wnk.1 - STE kinases |
| EC-SNU1_contig_28753 | Sobic.007G073400.1 | LOC_Os08g10300.1 | SHR5-receptor-like kinase |
| EC-SNU1_contig_28813 | Sobic.006G215600.1 | LOC_Os04g52600.1 | SHR5-receptor-like kinase |
| EC-SNU1_contig_29463 | Sobic.010G245100.1 | LOC_Os08g03240.1 | Lectin-like receptor kinase 1 |
| EC-SNU1_contig_29493 | Sobic.008G055400.1 | LOC_Os10g12620.1 | Protein kinase domain containing protein |
| EC-SNU1_contig_29499 | Sobic.004G214900.1 | LOC_Os02g40240.1 | Receptor kinase |
| EC-SNU1_contig_29724 | Sobic.005G122400.1 | LOC_Os11g07225.1 | protein |
| EC-SNU1_contig_29908 | Sobic.007G225900.1 | LOC_Os02g41500.1 | OsWAK13 receptor-like protein kinase |
| EC-SNU1_contig_29997 | Sobic.004G214900.1 | LOC_Os02g40240.1 | Receptor kinase |
| EC-SNU1_contig_425 | Sobic.001G236900.1 | LOC_Os10g30540.1 | Lectin-like receptor kinase |
| EC-SNU1_contig_29998 | Sobic.010G176700.1 | LOC_Os06g38670.1 | Receptor-like protein kinase precursor |
| EC-SNU1_contig_30038 | Sobic.008G022300.1 | LOC_Os11g35120.1 | OsWAK116 receptor-like cytoplasmic kinase OsWAK-RLCK |
| EC-SNU1_contig_30042 | Sobic.008G099300.1 | LOC_Os11g39450.1 | Cysteine-rich receptor-like protein kinase 7 precursor |
| EC-SNU1_contig_30095 | Sobic.005G038800.1 | LOC_Os07g03000.1 | Receptor-like protein kinase precursor |
| EC-SNU1_contig_30359 | Sobic.008G025600.1 | LOC_Os01g67160.1 | Cyclin-dependent kinase B1-1 |
| EC-SNU1_contig_30517 | N/A | N/A | Unknown |
| EC-SNU1_contig_30601 | N/A | N/A | Unknown |
| EC-SNU1_contig_611 | Sobic.001G168400.1 | LOC_Os03g41460.1 | CAMK_like.20 - calcium/calmodulin depedent protein kinases |
| EC-SNU1_contig_627 | Sobic.003G013400.1 | LOC_Os01g12290.1 | S-locus lectin protein kinase family protein |
| EC-SNU1_contig_30629 | Sobic.005G061500.4 | LOC_Os01g65330.1 | protein |
| EC-SNU1_contig_667 | Sobic.002G013800.3 | LOC_Os02g18080.1 | NB-ARC domain containing protein |
| EC-SNU1_contig_30729 | Sobic.003G024400.1 | LOC_Os01g10890.1 | CAMK_like.8 - calcium/calmodulin depedent protein kinases |
| EC-SNU1_contig_906 | Sobic.002G431600.2 | LOC_Os07g49470.1 | Protein kinase APK1B chloroplast precursor |
| EC-SNU1_contig_907 | Sobic.002G431600.2 | LOC_Os07g49470.1 | Protein kinase APK1B chloroplast precursor |
| EC-SNU1_contig_978 | Sobic.002G249700.1 | LOC_Os09g29510.1 | OsWAK80 receptor-like protein kinase |
| EC-SNU1_contig_31089 | Sobic.002G327500.1 | LOC_Os07g35290.1 | TKL_IRAK_DUF26-lc.10 - DUF26 kinases |
| EC-SNU1_contig_1442 | Sobic.004G217500.1 | LOC_Os02g40180.1 | Receptor-like protein kinase 5 precursor |
| EC-SNU1_contig_1472 | Sobic.008G151000.1 | LOC_Os04g20680.1 | Wall-associated receptor kinase 3 precursor |
| EC-SNU1_contig_1473 | Sobic.008G151000.1 | LOC_Os04g20680.1 | Wall-associated receptor kinase 3 precursor |
| EC-SNU1_contig_31168 | Sobic.008G007400.1 | LOC_Os11g47180.1 | Receptor-like protein kinase 2 precursor |
| EC-SNU1_contig_1682 | Sobic.004G214900.1 | LOC_Os02g40240.1 | Receptor kinase |
| EC-SNU1_contig_1766 | Sobic.002G249600.1 | LOC_Os09g29510.1 | OsWAK80 receptor-like protein kinase |
| EC-SNU1_contig_1837 | Sobic.009G082700.1 | LOC_Os04g01310.1 | Serine/threonine-protein kinase receptor precursor |
