# Supplementary material for: Uncovering the Differential Molecular Basis of Adaptive Diversity in Three Echinochloa Leaf Transcriptomes
Source: PLoS One. 2015 Aug 12;10(8):e0134419. doi: 10.1371/journal.pone.0134419 (PMC4534374; doi:10.1371/journal.pone.0134419)
Supplement: S6 Table — (DOCX) [file pone.0134419.s015.docx]

**S6 Table.** Hierarchical clustering of DEGs belonging to leucine-rich repeat protein kinase (LRR-STKs).

| **Contig ID** | ***S. bicolor* homolog ID** | ***O. sativa* homolog ID** | **Annotation** |
| --- | --- | --- | --- |
| EC-SNU1_contig_2311 | Sobic.004G214800.1 | LOC_Os02g40180.1 | Receptor-like protein kinase 5 precursor |
| EC-SNU1_contig_2312 | Sobic.010G020600.1 | LOC_Os06g03970.1 | Receptor-like protein kinase 5 precursor |
| EC-SNU1_contig_2619 | Sobic.009G040300.1 | LOC_Os02g40240.1 | Receptor kinase |
| EC-SNU1_contig_2620 | Sobic.004G214900.1 | LOC_Os02g40240.1 | Receptor kinase |
| EC-SNU1_contig_2661 | Sobic.008G132000.1 | LOC_Os12g37770.1 | RGH1A |
| EC-SNU1_contig_2679 | Sobic.004G214900.1 | LOC_Os02g40240.1 | Receptor kinase |
| EC-SNU1_contig_2869 | Sobic.005G228300.1 | LOC_Os05g16200.1 | Blight resistance protein T118 |
| EC-SNU1_contig_2871 | Sobic.005G228300.1 | LOC_Os05g16200.1 | Blight resistance protein T118 |
| EC-SNU1_contig_3145 | Sobic.007G183500.1 | LOC_Os08g43000.1 | CC-NBS-LRR |
| EC-SNU1_contig_3146 | N/A | N/A | Unknown |
| EC-SNU1_contig_3361 | Sobic.006G215400.1 | LOC_Os04g52590.1 | Protein kinase domain containing protein |
| EC-SNU1_contig_3477 | Sobic.003G067600.1 | LOC_Os01g05960.1 | Receptor kinase |
| EC-SNU1_contig_3626 | Sobic.002G013800.3 | LOC_Os02g18080.1 | NB-ARC domain containing protein |
| EC-SNU1_contig_4484 | Sobic.002G077400.1 | LOC_Os10g33040.1 | Receptor-like protein kinase precursor |
| EC-SNU1_contig_4551 | N/A | N/A | Unknown |
| EC-SNU1_contig_4552 | Sobic.004G214900.1 | LOC_Os02g40240.1 | Receptor kinase |
| EC-SNU1_contig_4657 | Sobic.007G144600.1 | LOC_Os08g34640.1 | Receptor-like protein kinase precursor |
| EC-SNU1_contig_4658 | Sobic.007G144700.1 | LOC_Os08g34640.1 | Receptor-like protein kinase precursor |
| EC-SNU1_contig_4659 | Sobic.007G144700.1 | LOC_Os08g34640.1 | Receptor-like protein kinase precursor |
| EC-SNU1_contig_4842 | Sobic.008G139100.1 | LOC_Os12g17480.1 | MLA12 |
| EC-SNU1_contig_4875 | Sobic.005G195000.2 | LOC_Os12g37770.1 | RGH1A |
| EC-SNU1_contig_4949 | Sobic.007G188800.1 | LOC_Os08g43000.1 | CC-NBS-LRR |
| EC-SNU1_contig_5417 | Sobic.002G013800.3 | LOC_Os02g18080.1 | NB-ARC domain containing protein |
| EC-SNU1_contig_5514 | N/A | N/A | Unknown |
| EC-SNU1_contig_6222 | Sobic.002G013800.3 | LOC_Os02g18080.1 | NB-ARC domain containing protein |
| EC-SNU1_contig_6413 | N/A | N/A | Unknown |
| EC-SNU1_contig_6415 | Sobic.005G095700.2 | LOC_Os11g11990.1 | NB-ARC domain containing protein |
| EC-SNU1_contig_7541 | Sobic.002G177800.1 | LOC_Os09g18360.1 | Expressed protein |
| EC-SNU1_contig_7543 | Sobic.002G177800.1 | LOC_Os09g18360.1 | Expressed protein |
| EC-SNU1_contig_8397 | N/A | N/A | Unknown |
| EC-SNU1_contig_8910 | Sobic.004G217500.1 | LOC_Os02g40180.1 | Receptor-like protein kinase 5 precursor |
| EC-SNU1_contig_9222 | Sobic.005G167500.1 | LOC_Os08g42670.1 | Resistance protein |
| EC-SNU1_contig_10372 | N/A | N/A | Unknown |
| EC-SNU1_contig_10373 | Sobic.008G131900.1 | LOC_Os12g37740.1 | Resistance protein LR10 |
| EC-SNU1_contig_12868 | Sobic.003G277900.1 | LOC_Os01g52050.1 | Systemin receptor SR160 precursor |
| EC-SNU1_contig_13999 | Sobic.005G212500.1 | LOC_Os11g45180.1 | NBS-LRR disease resistance protein |
| EC-SNU1_contig_14000 | Sobic.005G212500.1 | LOC_Os11g45180.1 | NBS-LRR disease resistance protein |
| EC-SNU1_contig_15026 | Sobic.002G128300.1 | LOC_Os01g21240.1 | MLA6 protein |
| EC-SNU1_contig_15989 | Sobic.001G202300.1 | LOC_Os10g35920.1 | OsFBX389 - F-box domain containing protein |
| EC-SNU1_contig_18930 | Sobic.005G219900.1 | LOC_Os11g46210.1 | NB-ARC domain containing protein |
| EC-SNU1_contig_19137 | Sobic.005G195000.2 | LOC_Os12g37770.1 | RGH1A |
| EC-SNU1_contig_23857 | Sobic.008G027200.1 | LOC_Os01g57310.1 | RP1 |
| EC-SNU1_contig_25790 | Sobic.K006100.1 | LOC_Os10g22980.1 | Leucine rich repeat domain containing protein |
| EC-SNU1_contig_28753 | Sobic.007G073400.1 | LOC_Os08g10300.1 | SHR5-receptor-like kinase |
| EC-SNU1_contig_28813 | Sobic.006G215600.1 | LOC_Os04g52600.1 | SHR5-receptor-like kinase |
| EC-SNU1_contig_29181 | Sobic.002G031100.2 | LOC_Os07g27370.1 | Resistance protein |
| EC-SNU1_contig_29431 | Sobic.010G164600.1 | LOC_Os08g14850.1 | Resistance protein |
| EC-SNU1_contig_29499 | Sobic.004G214900.1 | LOC_Os02g40240.1 | Receptor kinase |
| EC-SNU1_contig_29553 | Sobic.009G041400.1 | LOC_Os04g02030.1 | RP1 |
| EC-SNU1_contig_29573 | Sobic.002G013800.3 | LOC_Os02g18080.1 | NB-ARC domain containing protein |
| EC-SNU1_contig_36 | Sobic.006G146500.1 | LOC_Os04g43440.1 | NB-ARC/LRR disease resistance protein |
| EC-SNU1_contig_29724 | Sobic.005G122400.1 | LOC_Os11g07225.1 | Expressed protein |
| EC-SNU1_contig_403 | Sobic.005G182100.1 | LOC_Os11g39290.1 | Leucine Rich Repeat family protein |
| EC-SNU1_contig_29872 | Sobic.003G329300.1 | LOC_Os05g41290.1 | Disease resistance RPP13-like protein 1 |
| EC-SNU1_contig_29997 | Sobic.004G214900.1 | LOC_Os02g40240.1 | Receptor kinase |
| EC-SNU1_contig_425 | Sobic.001G236900.1 | LOC_Os10g30540.1 | Lectin-like receptor kinase |
| EC-SNU1_contig_29998 | Sobic.010G176700.1 | LOC_Os06g38670.1 | Receptor-like protein kinase precursor |
| EC-SNU1_contig_426 | Sobic.006G146500.1 | LOC_Os04g43440.1 | NB-ARC/LRR disease resistance protein |
| EC-SNU1_contig_30095 | Sobic.005G038800.1 | LOC_Os07g03000.1 | Receptor-like protein kinase precursor |
| EC-SNU1_contig_30344 | Sobic.007G186600.1 | LOC_Os08g42670.1 | Resistance protein |
| EC-SNU1_contig_30378 | Sobic.006G222900.1 | LOC_Os06g49390.1 | NBS-LRR disease resistance protein |
| EC-SNU1_contig_30601 | N/A | N/A | Unknown |
| EC-SNU1_contig_681 | Sobic.008G157400.1 | LOC_Os02g18000.1 | Disease resistance protein RGA2 |
| EC-SNU1_contig_30712 | Sobic.005G075800.1 | LOC_Os05g23990.1 | RP3 protein |
| EC-SNU1_contig_714 | Sobic.008G132000.1 | LOC_Os12g37770.1 | RGH1A |
| EC-SNU1_contig_30786 | Sobic.002G013800.3 | LOC_Os02g18080.1 | NB-ARC domain containing protein |
| EC-SNU1_contig_30841 | Sobic.006G146500.1 | LOC_Os04g43440.1 | NB-ARC/LRR disease resistance protein |
| EC-SNU1_contig_31015 | Sobic.005G076100.1 | LOC_Os05g23990.1 | RP3 protein |
| EC-SNU1_contig_31094 | Sobic.005G192100.2 | LOC_Os11g34920.1 | Stripe rust resistance protein Yr10 |
| EC-SNU1_contig_1339 | Sobic.005G222600.1 | LOC_Os11g43700.1 | RGH1A |
| EC-SNU1_contig_1442 | Sobic.004G217500.1 | LOC_Os02g40180.1 | Receptor-like protein kinase 5 precursor |
| EC-SNU1_contig_31168 | Sobic.008G007400.1 | LOC_Os11g47180.1 | Receptor-like protein kinase 2 precursor |
| EC-SNU1_contig_1682 | Sobic.004G214900.1 | LOC_Os02g40240.1 | Receptor kinase |
| EC-SNU1_contig_163 | Sobic.010G176700.1 | LOC_Os06g38670.1 | Receptor-like protein kinase precursor |
| EC-SNU1_contig_1852 | Sobic.007G189200.1 | LOC_Os08g43000.1 | CC-NBS-LRR |
