# Supplementary material for: Uncovering the Differential Molecular Basis of Adaptive Diversity in Three Echinochloa Leaf Transcriptomes
Source: PLoS One. 2015 Aug 12;10(8):e0134419. doi: 10.1371/journal.pone.0134419 (PMC4534374; doi:10.1371/journal.pone.0134419)
Supplement: S7 Table — (DOCX) [file pone.0134419.s016.docx]

**S7 Table.** Hierarchical clustering of DEGs belonging to calcium-related signaling.

| **Contig ID** | ***A. thaliana* homolog** | **Annotation** |
| --- | --- | --- |
| EC-SNU1_contig_12666 | AT3G47480 | Calcium-binding EF-hand family protein |
| EC-SNU1_contig_13763 | AT5G58380 | SOS3-interacting protein 1 |
| EC-SNU1_contig_14184 | AT1G64850 | Calcium-binding EF hand family protein |
| EC-SNU1_contig_14757 | AT1G24620 | EF hand calcium-binding protein family |
| EC-SNU1_contig_14759 | AT1G24620 | EF hand calcium-binding protein family |
| EC-SNU1_contig_17711 | AT2G17290 | Calcium-dependent protein kinase family protein |
| EC-SNU1_contig_17715 | AT2G17290 | Calcium-dependent protein kinase family protein |
| EC-SNU1_contig_17727 | AT2G17290 | Calcium-dependent protein kinase family protein |
| EC-SNU1_contig_17731 | AT2G17290 | Calcium-dependent protein kinase family protein |
| EC-SNU1_contig_19339 | AT2G46600 | Calcium-binding EF-hand family protein |
| EC-SNU1_contig_22809 | AT4G23650 | Calcium-dependent protein kinase 6 |
| EC-SNU1_contig_22813 | AT4G23650 | Calcium-dependent protein kinase 6 |
| EC-SNU1_contig_23552 | AT4G32060 | Calcium-binding EF hand family protein |
| EC-SNU1_contig_23554 | AT4G32060 | Calcium-binding EF hand family protein |
| EC-SNU1_contig_24035 | AT3G17510 | CBL-interacting protein kinase 1 |
| EC-SNU1_contig_24038 | AT1G48260 | CBL-interacting protein kinase 17 |
| EC-SNU1_contig_24131 | AT5G04870 | Calcium dependent protein kinase 1 |
| EC-SNU1_contig_24723 | AT2G17290 | Calcium-dependent protein kinase family protein |
| EC-SNU1_contig_25082 | AT2G26980 | CBL-interacting protein kinase 3 |
| EC-SNU1_contig_25090 | AT5G21326 | Ca2+regulated serine-threonine protein kinase family protein |
| EC-SNU1_contig_25091 | AT5G21326 | Ca2+regulated serine-threonine protein kinase family protein |
| EC-SNU1_contig_25094 | AT2G26980 | CBL-interacting protein kinase 3 |
| EC-SNU1_contig_29736 | AT5G37780 | Calmodulin 1 |
| EC-SNU1_contig_30729 | AT5G58380 | SOS3-interacting protein 1 |
