# Supplementary material for: Uncovering the Differential Molecular Basis of Adaptive Diversity in Three Echinochloa Leaf Transcriptomes
Source: PLoS One. 2015 Aug 12;10(8):e0134419. doi: 10.1371/journal.pone.0134419 (PMC4534374; doi:10.1371/journal.pone.0134419)
Supplement: S8 Table — (DOCX) [file pone.0134419.s017.docx]

**S8 Table**. Hierarchical clustering of DEGs belonging to signaling-related proteins.

| **Contig ID** | ***S. bicolor* homolog ID** | ***O. sativa* homolog ID** | **Annotation** |
| --- | --- | --- | --- |
| EC-SNU1_contig_2312 | Sobic.010G020600.1 | LOC_Os06g03970.1 | Receptor-like protein kinase 5 precursor |
| EC-SNU1_contig_2476 | Sobic.005G096400.1 | LOC_Os11g11890.1 | Protein kinase domain containing protein |
| EC-SNU1_contig_3057 | N/A | N/A | Unknown |
| EC-SNU1_contig_3358 | Sobic.003G421800.1 | LOC_Os01g71420.1 | Ser/Thr protein phosphatase family protein |
| EC-SNU1_contig_3359 | Sobic.003G421800.1 | LOC_Os01g71420.1 | Ser/Thr protein phosphatase family protein |
| EC-SNU1_contig_3361 | Sobic.006G215400.1 | LOC_Os04g52590.1 | Protein kinase domain containing protein |
| EC-SNU1_contig_4484 | Sobic.002G077400.1 | LOC_Os10g33040.1 | Receptor-like protein kinase precursor |
| EC-SNU1_contig_4552 | Sobic.004G214900.1 | LOC_Os02g40240.1 | Receptor kinase |
| EC-SNU1_contig_5078 | Sobic.008G189500.1 | LOC_Os12g44020.1 | Ser/Thr protein phosphatase family protein |
| EC-SNU1_contig_5973 | Sobic.007G075700.1 | LOC_Os08g10630.1 | Metal cation transporter |
| EC-SNU1_contig_6297 | Sobic.004G305000.1 | LOC_Os02g52210.1 | Zinc finger, C3HC4 type domain containing protein |
| EC-SNU1_contig_6362 | Sobic.005G169300.1 | LOC_Os11g37970.1 | WIP5 - Wound-induced protein precursor |
| EC-SNU1_contig_6363 | Sobic.005G112700.1 | LOC_Os11g37970.1 | WIP5 - Wound-induced protein precursor |
| EC-SNU1_contig_6365 | N/A | N/A | Unknown |
| EC-SNU1_contig_7183 | Sobic.004G165300.1 | LOC_Os02g32980.1 | Cupin domain containing protein |
| EC-SNU1_contig_7370 | N/A | N/A | Unknown |
| EC-SNU1_contig_7518 | Sobic.004G219700.1 | LOC_Os02g41500.1 | OsWAK13 receptor-like protein kinase |
| EC-SNU1_contig_7541 | Sobic.002G177800.1 | LOC_Os09g18360.1 | Expressed protein |
| EC-SNU1_contig_7543 | Sobic.002G177800.1 | LOC_Os09g18360.1 | Expressed protein |
| EC-SNU1_contig_7551 | Sobic.008G030600.1 | LOC_Os12g02340.1 | Protease inhibitor/seed storage/LTP family protein precursor |
| EC-SNU1_contig_10605 | Sobic.004G170600.1 | LOC_Os06g06260.1 | GDSL-like lipase/acylhydrolase |
| EC-SNU1_contig_11380 | Sobic.006G205600.1 | LOC_Os04g51460.1 | Glycosyl hydrolases family 16 |
| EC-SNU1_contig_11383 | Sobic.006G205600.1 | LOC_Os04g51460.1 | Glycosyl hydrolases family 16 |
| EC-SNU1_contig_11384 | Sobic.006G205600.1 | LOC_Os04g51460.1 | Glycosyl hydrolases family 16 |
| EC-SNU1_contig_11385 | Sobic.006G205600.1 | LOC_Os04g51460.1 | Glycosyl hydrolases family 16 |
| EC-SNU1_contig_11386 | Sobic.006G205600.1 | LOC_Os04g51460.1 | Glycosyl hydrolases family 16 |
| EC-SNU1_contig_11722 | Sobic.005G063600.1 | LOC_Os11g11890.1 | Protein kinase domain containing protein |
| EC-SNU1_contig_11727 | Sobic.005G063600.1 | LOC_Os11g11890.1 | Protein kinase domain containing protein |
| EC-SNU1_contig_12458 | Sobic.010G044500.2 | LOC_Os06g06250.2 | GDSL-like lipase/acylhydrolase |
| EC-SNU1_contig_12459 | Sobic.009G078500.1 | LOC_Os05g11910.1 | GDSL-like lipase/acylhydrolase |
| EC-SNU1_contig_12627 | Sobic.008G163200.1 | LOC_Os02g46260.1 | OsSCP9 - Putative Serine Carboxypeptidase homologue |
| EC-SNU1_contig_12629 | Sobic.008G163200.1 | LOC_Os02g46260.1 | OsSCP9 - Putative Serine Carboxypeptidase homologue |
| EC-SNU1_contig_12634 | Sobic.008G163200.1 | LOC_Os02g46260.1 | OsSCP9 - Putative Serine Carboxypeptidase homologue |
| EC-SNU1_contig_12622 | Sobic.008G163200.1 | LOC_Os02g46260.1 | OsSCP9 - Putative Serine Carboxypeptidase homologue |
| EC-SNU1_contig_12638 | Sobic.008G163200.2 | LOC_Os02g46260.2 | OsSCP9 - Putative Serine Carboxypeptidase homologue |
| EC-SNU1_contig_12750 | Sobic.009G059100.1 | LOC_Os08g35760.1 | Cupin domain containing protein |
| EC-SNU1_contig_12868 | Sobic.003G277900.1 | LOC_Os01g52050.1 | Systemin receptor SR160 precursor |
| EC-SNU1_contig_13317 | N/A | N/A | Unknown |
| EC-SNU1_contig_13318 | Sobic.010G246700.1 | LOC_Os06g48200.1 | Glycosyl hydrolases family 16 |
| EC-SNU1_contig_13319 | Sobic.010G246400.1 | LOC_Os06g48160.1 | Glycosyl hydrolases family 16 |
| EC-SNU1_contig_14389 | Sobic.001G113800.1 | LOC_Os03g50810.1 | Receptor protein kinase TMK1 precursor |
| EC-SNU1_contig_14990 | Sobic.001G516500.1 | LOC_Os03g04020.1 | Expansin precursor |
| EC-SNU1_contig_15643 | Sobic.002G329100.1 | LOC_Os07g35750.1 | DUF26 kinases have homology to DUF26 containing loci |
| EC-SNU1_contig_15644 | N/A | N/A | Unknown |
| EC-SNU1_contig_18088 | Sobic.004G273200.1 | LOC_Os02g46910.1 | Glycosyl hydrolases family 16 |
| EC-SNU1_contig_18089 | Sobic.004G273200.1 | LOC_Os02g46910.1 | Glycosyl hydrolases family 16 |
| EC-SNU1_contig_18563 | N/A | N/A | Unknown |
| EC-SNU1_contig_20408 | Sobic.003G218900.1 | LOC_Os01g42730.1 | GDSL-like lipase/acylhydrolase |
| EC-SNU1_contig_20411 | Sobic.003G218900.1 | LOC_Os01g42730.1 | GDSL-like lipase/acylhydrolase |
| EC-SNU1_contig_20567 | Sobic.008G017800.1 | LOC_Os11g03730.1 | Alpha-N-arabinofuranosidase A |
| EC-SNU1_contig_20570 | Sobic.005G087400.1 | LOC_Os11g03730.1 | Alpha-N-arabinofuranosidase A |
| EC-SNU1_contig_20578 | Sobic.008G017800.1 | LOC_Os11g03730.1 | Alpha-N-arabinofuranosidase A |
| EC-SNU1_contig_20568 | Sobic.005G087400.1 | LOC_Os11g03730.1 | Alpha-N-arabinofuranosidase A |
| EC-SNU1_contig_20583 | Sobic.008G017800.1 | LOC_Os11g03730.1 | Alpha-N-arabinofuranosidase A |
| EC-SNU1_contig_20585 | Sobic.005G087400.1 | LOC_Os11g03730.1 | Alpha-N-arabinofuranosidase A |
| EC-SNU1_contig_20588 | Sobic.008G017800.1 | LOC_Os11g03730.1 | Alpha-N-arabinofuranosidase A |
| EC-SNU1_contig_20590 | Sobic.008G017800.1 | LOC_Os11g03730.1 | Alpha-N-arabinofuranosidase A |
| EC-SNU1_contig_20591 | Sobic.008G017800.1 | LOC_Os11g03730.1 | Alpha-N-arabinofuranosidase A |
| EC-SNU1_contig_20593 | Sobic.005G087400.1 | LOC_Os11g03730.1 | Alpha-N-arabinofuranosidase A |
| EC-SNU1_contig_20597 | Sobic.005G087400.1 | LOC_Os11g03730.1 | Alpha-N-arabinofuranosidase A |
| EC-SNU1_contig_21676 | Sobic.006G145700.1 | LOC_Os04g43360.1 | Os4bglu14 - monolignol beta-glucoside homologue |
| EC-SNU1_contig_21689 | Sobic.001G399900.1 | LOC_Os03g18910.1 | COBRA-like protein 7 precursor |
| EC-SNU1_contig_22503 | Sobic.006G071400.1 | LOC_Os08g13920.1 | Glycosyl hydrolases family 16 |
| EC-SNU1_contig_22499 | Sobic.006G071400.1 | LOC_Os08g13920.1 | Glycosyl hydrolases family 16 |
| EC-SNU1_contig_22501 | Sobic.006G070600.1 | LOC_Os08g13920.1 | Glycosyl hydrolases family 16 |
| EC-SNU1_contig_23095 | Sobic.007G190200.2 | LOC_Os08g41880.1 | Nucleotide pyrophosphatase/phosphodiesterase |
| EC-SNU1_contig_23096 | Sobic.007G190200.1 | LOC_Os08g41880.1 | Nucleotide pyrophosphatase/phosphodiesterase |
| EC-SNU1_contig_23706 | Sobic.010G173800.1 | LOC_Os06g37560.1 | Beta-galactosidase precursor |
| EC-SNU1_contig_26617 | Sobic.009G114000.1 | LOC_Os05g30350.1 | Os5bglu22 - beta-glucosidase homologue |
| EC-SNU1_contig_27289 | Sobic.003G332700.3 | LOC_Os01g70520.1 | Os1bglu5 - beta-glucosidase homologue |
| EC-SNU1_contig_27294 | Sobic.003G332700.1 | LOC_Os01g70520.1 | Os1bglu5 - beta-glucosidase homologue |
| EC-SNU1_contig_27296 | N/A | N/A | Unknown |
| EC-SNU1_contig_27298 | Sobic.003G332700.3 | LOC_Os01g70520.1 | Os1bglu5 - beta-glucosidase homologue |
| EC-SNU1_contig_28753 | Sobic.007G073400.1 | LOC_Os08g10300.1 | SHR5-receptor-like kinase |
| EC-SNU1_contig_316 | Sobic.003G013100.1 | LOC_Os01g12320.1 | GDSL-like lipase/acylhydrolase |
| EC-SNU1_contig_29277 | Sobic.010G246400.1 | LOC_Os06g48160.1 | Glycosyl hydrolases family 16 |
| EC-SNU1_contig_29499 | Sobic.004G214900.1 | LOC_Os02g40240.1 | Receptor kinase |
| EC-SNU1_contig_29724 | Sobic.005G122400.1 | LOC_Os11g07225.1 | Expressed protein |
| EC-SNU1_contig_29908 | Sobic.007G225900.1 | LOC_Os02g41500.1 | OsWAK13 - OsWAK receptor-like protein kinase |
| EC-SNU1_contig_29998 | Sobic.010G176700.1 | LOC_Os06g38670.1 | Receptor-like protein kinase precursor |
| EC-SNU1_contig_30042 | Sobic.008G099300.1 | LOC_Os11g39450.1 | Cysteine-rich receptor-like protein kinase 7 precursor |
| EC-SNU1_contig_30192 | Sobic.008G080100.1 | LOC_Os04g39880.1 | Os4bglu12 - beta-glucosidase, exo-beta-glucanase |
| EC-SNU1_contig_667 | Sobic.002G013800.3 | LOC_Os02g18080.1 | NB-ARC domain containing protein |
| EC-SNU1_contig_30883 | Sobic.001G529700.1 | LOC_Os03g02750.1 | OsSub25 - Putative Subtilisin homologue |
| EC-SNU1_contig_868 | Sobic.008G079800.1 | LOC_Os04g39880.1 | Os4bglu12 - beta-glucosidase, exo-beta-glucanase |
| EC-SNU1_contig_1008 | Sobic.003G422000.1 | LOC_Os01g71670.1 | Glycosyl hydrolases family 17 |
| EC-SNU1_contig_31089 | Sobic.002G327500.1 | LOC_Os07g35290.1 | DUF26 kinases have homology to DUF26 containing loci |
| EC-SNU1_contig_1442 | Sobic.004G217500.1 | LOC_Os02g40180.1 | Receptor-like protein kinase 5 precursor |
| EC-SNU1_contig_1472 | Sobic.008G151000.1 | LOC_Os04g20680.1 | Wall-associated receptor kinase 3 precursor |
| EC-SNU1_contig_1473 | Sobic.008G151000.1 | LOC_Os04g20680.1 | Wall-associated receptor kinase 3 precursor |
| EC-SNU1_contig_31168 | Sobic.008G007400.1 | LOC_Os11g47180.1 | Receptor-like protein kinase 2 precursor |
