# Supplementary material for: Uncovering the Differential Molecular Basis of Adaptive Diversity in Three Echinochloa Leaf Transcriptomes
Source: PLoS One. 2015 Aug 12;10(8):e0134419. doi: 10.1371/journal.pone.0134419 (PMC4534374; doi:10.1371/journal.pone.0134419)
Supplement: S10 Table — (DOCX) [file pone.0134419.s019.docx]

**S10 Table.** Hierarchical clustering of DEGs belonging to glycosidases.

| **Contig ID** | ***S. bicolor* homolog ID** | ***O. sativa* homolog ID** | **Annotation** |
| --- | --- | --- | --- |
| EC-SNU1_contig_7049 | N/A | N/A | Unknown |
| EC-SNU1_contig_11383 | Sobic.006G205600.1 | LOC_Os04g51460.1 | Glycosyl hydrolases family 16 |
| EC-SNU1_contig_11384 | Sobic.006G205600.1 | LOC_Os04g51460.1 | Glycosyl hydrolases family 16 |
| EC-SNU1_contig_11385 | Sobic.006G205600.1 | LOC_Os04g51460.1 | Glycosyl hydrolases family 16 |
| EC-SNU1_contig_11386 | Sobic.006G205600.1 | LOC_Os04g51460.1 | Glycosyl hydrolases family 16 |
| EC-SNU1_contig_11626 | N/A | N/A | Unknown |
| EC-SNU1_contig_11627 | Sobic.004G208700.1 | LOC_Os02g39330.1 | CHIT1 - Chitinase family protein precursor |
| EC-SNU1_contig_13317 | N/A | N/A | Unknown |
| EC-SNU1_contig_13318 | Sobic.010G246700.1 | LOC_Os06g48200.1 | Glycosyl hydrolases family 16 |
| EC-SNU1_contig_13319 | Sobic.010G246400.1 | LOC_Os06g48160.1 | Glycosyl hydrolases family 16 |
| EC-SNU1_contig_15324 | Sobic.002G328300.1 | LOC_Os07g35350.1 | Glucan endo-1,3-beta-glucosidase precursor |
| EC-SNU1_contig_15326 | Sobic.002G327900.2 | LOC_Os08g14700.1 | Glucan endo-1,3-beta-glucosidase precursor |
| EC-SNU1_contig_18088 | Sobic.004G273200.1 | LOC_Os02g46910.1 | Glycosyl hydrolases family 16 |
| EC-SNU1_contig_18089 | Sobic.004G273200.1 | LOC_Os02g46910.1 | Glycosyl hydrolases family 16 |
| EC-SNU1_contig_21676 | Sobic.006G145700.1 | LOC_Os04g43360.1 | Os4bglu14 - monolignol beta-glucoside homologue |
| EC-SNU1_contig_21872 | Sobic.004G004800.1 | LOC_Os02g01590.1 | Glycosyl hydrolases |
| EC-SNU1_contig_22503 | Sobic.006G071400.1 | LOC_Os08g13920.1 | Glycosyl hydrolases family 16 |
| EC-SNU1_contig_22499 | Sobic.006G071400.1 | LOC_Os08g13920.1 | Glycosyl hydrolases family 16 |
| EC-SNU1_contig_22501 | Sobic.006G070600.1 | LOC_Os08g13920.1 | Glycosyl hydrolases family 16 |
| EC-SNU1_contig_27294 | Sobic.003G332700.1 | LOC_Os01g70520.1 | Os1bglu5 - beta-glucosidase homologue |
| EC-SNU1_contig_27296 | N/A | N/A | Unknown |
| EC-SNU1_contig_29277 | Sobic.010G246400.1 | LOC_Os06g48160.1 | Glycosyl hydrolases family 16 |
| EC-SNU1_contig_30192 | Sobic.008G080100.1 | LOC_Os04g39880.1 | Os4bglu12 - beta-glucosidase, exo-beta-glucanase |
| EC-SNU1_contig_868 | Sobic.008G079800.1 | LOC_Os04g39880.1 | Os4bglu12 - beta-glucosidase, exo-beta-glucanase |
