# Supplementary material for: Uncovering the Differential Molecular Basis of Adaptive Diversity in Three Echinochloa Leaf Transcriptomes
Source: PLoS One. 2015 Aug 12;10(8):e0134419. doi: 10.1371/journal.pone.0134419 (PMC4534374; doi:10.1371/journal.pone.0134419)
Supplement: S11 Table — (DOCX) [file pone.0134419.s020.docx]

**S11 Table.** Hierarchical clustering of DEGs belonging to previously known *Arabidopsis* abiotic stress-responsive genes.

| **Contig ID** | ***A. thaliana* homolog** | **Annotation** |
| --- | --- | --- |
| EC-SNU1_contig_9337 | AT3G23400 | Plastid-lipid associated protein PAP / fibrillin family protein |
| EC-SNU1_contig_6618 | AT1G71860 | Protein tyrosine phosphatase 1 |
| EC-SNU1_contig_6627 | AT1G71860 | Protein tyrosine phosphatase 1 |
| EC-SNU1_contig_6620 | AT1G71860 | Protein tyrosine phosphatase 1 |
| EC-SNU1_contig_6628 | AT1G71860 | Protein tyrosine phosphatase 1 |
| EC-SNU1_contig_6624 | AT1G71860 | Protein tyrosine phosphatase 1 |
| EC-SNU1_contig_6631 | AT1G71860 | Protein tyrosine phosphatase 1 |
| EC-SNU1_contig_6619 | AT1G71860 | Protein tyrosine phosphatase 1 |
| EC-SNU1_contig_15398 | AT2G45910 | U-box domain-containing protein kinase family protein |
| EC-SNU1_contig_6625 | AT1G71860 | Protein tyrosine phosphatase 1 |
| EC-SNU1_contig_14123 | AT5G44650 | Nucleus-encoded thylakoid protein |
| EC-SNU1_contig_8622 | AT5G50720 | HVA22 homologue E |
| EC-SNU1_contig_18563 | AT4G33420 | Peroxidase superfamily protein |
| EC-SNU1_contig_21769 | AT3G48170 | Aldehyde dehydrogenase 10A9 |
| EC-SNU1_contig_25712 | AT5G12010 | Unknown |
| EC-SNU1_contig_3057 | AT4G33420 | Peroxidase superfamily protein |
| EC-SNU1_contig_28685 | AT1G64550 | General control non-repressible 3 |
| EC-SNU1_contig_26452 | AT5G07350 | TUDOR-SN protein 1 |
| EC-SNU1_contig_5805 | AT2G04240 | RING/U-box superfamily protein |
| EC-SNU1_contig_6701 | AT1G22190 | Integrase-type DNA-binding superfamily protein |
| EC-SNU1_contig_30183 | AT3G01420 | Peroxidase superfamily protein |
| EC-SNU1_contig_12175 | AT2G19810 | CCCH-type zinc finger family protein |
| EC-SNU1_contig_15397 | AT2G45910 | U-box domain-containing protein kinase family protein |
| EC-SNU1_contig_21779 | AT1G53670 | Methionine sulfoxide reductase B 1 |
| EC-SNU1_contig_20949 | AT3G01650 | RING domain ligase1 |
| EC-SNU1_contig_15746 | AT3G23600 | Alpha/beta-Hydrolases superfamily protein |
| EC-SNU1_contig_21778 | AT1G53670 | Methionine sulfoxide reductase B 1 |
| EC-SNU1_contig_21176 | AT2G32500 | Stress responsive alpha-beta barrel domain protein |
| EC-SNU1_contig_13920 | AT5G56550 | Oxidative stress 3 |
| EC-SNU1_contig_25710 | AT5G12010 | Unknown |
| EC-SNU1_contig_25713 | AT5G12010 | Unknown |
| EC-SNU1_contig_25709 | AT5G12010 | Unknown |
| EC-SNU1_contig_12176 | AT2G19810 | CCCH-type zinc finger family protein |
| EC-SNU1_contig_22157 | AT2G38470 | WRKY DNA-binding protein 33 |
| EC-SNU1_contig_611 | AT3G50500 | SNF1-related protein kinase 2.2 |
| EC-SNU1_contig_9346 | AT3G05880 | Low temperature and salt responsive protein family |
| EC-SNU1_contig_20947 | AT3G01650 | RING domain ligase1 |
| EC-SNU1_contig_1454 | AT3G01420 | Peroxidase superfamily protein |
| EC-SNU1_contig_27924 | AT1G32230 | WWE protein-protein interaction domain protein family |
| EC-SNU1_contig_14930 | AT4G34180 | Cyclase family protein |
| EC-SNU1_contig_15744 | AT3G23600 | Alpha/beta-Hydrolases superfamily protein |
| EC-SNU1_contig_21780 | AT1G53670 | Methionine sulfoxide reductase B 1 |
| EC-SNU1_contig_6629 | AT1G71860 | Protein tyrosine phosphatase 1 |
| EC-SNU1_contig_6845 | AT1G23230 | Mediator complex subunit Med23 |
| EC-SNU1_contig_28444 | AT4G16760 | Acyl-CoA oxidase 1 |
| EC-SNU1_contig_28450 | AT4G16760 | Acyl-CoA oxidase 1 |
| EC-SNU1_contig_28445 | AT4G16760 | Acyl-CoA oxidase 1 |
| EC-SNU1_contig_1642 | AT1G75280 | NmrA-like negative transcriptional regulator family protein |
